# Supplementary material for: Central cavity dehydration as a gating mechanism of potassium channels
Source: Nat Commun. 2023 Apr 17;14:2178. doi: 10.1038/s41467-023-37531-8 (PMC10110622; doi:10.1038/s41467-023-37531-8)
Supplement: Supplementary file 1 — Supplementary Information [file 41467_2023_37531_MOESM1_ESM.pdf]

Supplementary information for

Central Cavity Dehydration as A Gating Mechanism of Potassium Channels

Authors: Gu et al.

This file includes:

Supplementary Notes 1-6

Supplementary Methods

Supplementary Tables 1-4

Supplementary Figures 1-27

Supplementary References 1-12

Supplementary Note 1 – Effects of Voltage and Ion Concentration on Ion Conduction. The number of water molecules in the cavities of the wild type and mutants were not affected by different voltages and ion concentrations (Supplementary Fig. 8f). However, the number of potassium ions in the cavities were reduced under lower voltage and ion concentration in most of the simulations, which are consistent with the reduced currents (Supplementary Fig. 8g). The number of potassium ions in the cavity were very low (< 1 in most simulations), except for the Ala305Glu mutant (both the open and closed states, between 2-5, depending on ion concentrations and voltages), in which the negatively charged Glu provided additional binding sites for potassium ions in the cavity.

It seemed that voltage had a larger impact on ion current than the potassium concentration in the cases tested in this work. For the simulations with open state backbones, reducing the ion concentration by 85% (from 1 M to 0.15 M) at 300 mV resulted ~20% decrease of the currents (Supplementary Fig. 8e), while the results at 150 mV for the wild type of the open state revealed ~40% decrease of the current (7.9 v.s. 4.6 pA, Supplementary Table 1). At 0.15 M, reducing the voltage from 300 mV to 150 mV (a 50% decrease) resulted ~65% decrease of the currents (Supplementary Fig. 8e). The results at 1 M for the wild type of the open state revealed ~50% decrease of the current (14.4 v.s. 7.9 pA, Supplementary Table 1). Therefore, the currents are overall more sensitive to the voltage than the ion concentration in the test range.

Effects of voltage and ion concentration on the currents may be different for the open and the closed states. At 0.15 M, reducing the voltage from 300 mV to 150 mV changed the currents of the open and closed states to similar extent (a ~65% decrease, as abovementioned), whereas at 300 mV, reducing the ion concentration from 1 M to 0.15 M resulted more significant decrease of the current for the closed state than the open state (Supplementary Fig. 8e). We hypothesize that the structure/shape of the cavity may be responsible for this difference, although a thorough test is beyond the scope of this work.

Supplementary Note 2 - Pro309 Also Plays a Role in Bending the Inner TM Helices. In addition to Gly302, Pro309 can also disrupt the backbone hydrogen bonds of α helices and resulted in a bending of the TM helices. Most hydrogen bonds around Pro309 showed bimodal distributions for their lengths (Supplementary Fig. 11). Correlations between the lengths of these hydrogen bonds revealed by 2D distributions indicated two states for the

inner TM helices in MD simulations (Supplementary Fig. 11). Simulations restraining these hydrogen bonds (Supplementary Table 2) revealed similar hydration levels, ion permeabilities and free energy barriers for the two states, although the inner TM helices had large bending in one state than the other (Supplementary Fig. 12).

Supplementary Note 3 - Effects of Gly302 and Pro309 on Channel Gating.

The kink of the inner TM helices was ascribed to a glycine, which is known to disrupt the conformation of α-helices. In this regard, we studied effects of Gly302Ala on channel gating.

The Gly302Ala mutant in equilibrium simulations was slightly more dehydrated and showed smaller ion current and larger energy barriers for ion and water permeation compared to the wild type in the closed state (water numbers of ~16 v.s. ~23, and ion currents of ~0.3 pA v.s. 1.3 pA, Supplementary Figs. 14-15, Supplementary Table 1). In enforced dewetting simulations starting from the open state, conformational transition showed much better convergence than the corresponding simulations of the wild type. Specifically, conformational changes from open to the closed state were induced successfully in all of the 10 replicas (success rate of 100%, see RMSD and water numbers in Fig. 4b-c). In addition, the resulted closed state is more similar to the structure in equilibrium simulations than the wild type case (RMSD of average structure over all replicas reduced from 0.12 nm in the wild type case to 0.10 nm in the mutant, see Supplementary Fig. 16, also see Fig. 4b). Moreover, distributions of hydrogen bonds indicated rewinding of the kink region for all subunits (Supplementary Fig. 17), whereas there was a minor fraction of failed rewinding in the wild type simulations (Fig. 4d).

In conclusion, these data suggest that the Gly302Ala mutation favors formation of the closed state as compared to the wild type, emphasizing the significance of a glycine at this position.

We also explored effects of Pro309Ala on channel gating, because previous analyses in Supplementary Fig. 11-12 indicated that the Pro309 plays a role in bending the inner TM helices and affects the conformation of the closed state.

The tetramer structures of the Pro309Ala mutant collapsed in 5 out of 10 equilibrium simulations. In these collapsed simulations, the tetramer arrangement of the inner TM helices, not the backbone hydrogen bonds of their α-helices, was deformed, implying the role of

helical bending around Pro309 for the tetramer structure. In the simulations with tetramer conformation maintained, we found lower hydration level (water number of ~7, Supplementary Fig. 14) and a much higher energy barrier for water entering into the cavity (~4 k_BT, Supplementary Fig.14), probably due to the expected straighter inner TM helices (see structural alignment in Supplementary Fig. 14 and hydrogen bond distributions in Supplementary Fig. 15). In enforced dewetting simulations starting from the open state, 19 out of the 20 replicas successfully resulted in closed state (see RMSD values and hydration levels in Fig. 4b-c). However, the average structures obtained in non-equilibrium and equilibrium simulations of the mutant had a moderate RMSD value of 0.20 nm (Supplementary Fig. 16, also see boxplot of RMSD in Fig. 4b), due to the imperfect rewinding of the kink region and a larger bending of the inner TM helices in the non-equilibrium simulations (see hydrogen bond distributions at the kink in Supplementary Fig. 17). There was a notable fraction of the subunits in which the kink was not rewound in the non-equilibrium simulations (Supplementary Fig. 17).

It seemed that, if the simulations were started from a structure lacking the kink (i.e., equilibrium simulations of Pro309Ala), a straightening of the inner helices tends to result in a collapse of the tetramer, while if the initial conformation has a kink (non-equilibrium simulations), a straightening of the helices around Ala309 prevents the kink from rewinding. We conclude that bending of the inner helices around Pro309 is essential for proper arrangement of the tetramer structure by avoiding steric hindrance between the four subunits.

Supplementary Note 4 – Cavity Hydration Induced Conformational Transition from the Closed to the Open State.

Conformational transitions from the closed to the open state were observed in the Ala305Glu mutant simulations discussed in the main text (Fig. 2, Supplementary Table 1). 0.2 μs simulations were first conducted for the mutant of the closed cryo-EM structure without voltage applied to equilibrate the system (referred as equilibrium simulations). Snapshots extracted randomly from the last 10 ns of these trajectories were used to continue the simulations for 0.5 μs (10 replicates) with a transmembrane voltage of 300 mV (referred as production simulations). Water numbers in the cavity (Supplementary Fig. 21b) and ion currents (Supplementary Table 1) suggested a fully hydrated cavity and a conductive channel similar to the open state. Boxplots of the RMSD values and the structural alignments of the

average structures (Supplementary Fig. 21a-b) indicated a trend of conformational transitions toward the open state. Detailed analyses of a representative simulation showed broken of the hydrogen bonds at the kink (297O-301HN and 298O-302HN) and shifting of the Phe304 sidechain toward the subunit interfaces, accompanied by a decrease of the RMSD values relative to the open state (Supplementary Fig. 21c-d), as expected. However, the average structure obtained based on all simulation replicas only showed moderate similarity to that of the open state equilibrium simulations (RMSD values of 0.19 and 0.17 nm, see Supplementary Fig. 21a). This is because not all of the four subunits underwent conformational transitions, as characterized by the hydrogen bonds at the kink of the inner TM helices. In the simulation shown in Supplementary Fig. 21c-d, the kink hydrogen bonds broke quickly in two of the subunits in the equilibrium simulation, while remaining stable in the other two subunits until the last 0.2 μs of the production simulation.

We also applied non-equilibrium simulations for the wild type channel to further explore cavity hydration induced conformational transitions. Specifically, the cavity of the closed cryo-EM structure was restrained to be hydrated for 0.5 μs simulations using the chain reaction coordinate (see Supplementary Methods section for details), which was then followed by another 0.5 μs simulation after removal of the restraints. We obtained partially hydrated structures, as suggested by the number of water molecules in the cavity (Supplementary Fig. 21b). Although the simulations did not fully converge to the open state (see the boxplot of the RMSD values in Supplementary Fig. 21b), they suggested a clear trend of conformational transition (see alignment of the average structures and the representative simulation in Supplementary Fig. 21a, c). In the representative simulation, one subunit underwent conformational transition within 0.2 μs, one subunit had partial unwinding of the kink region (as indicated by the very large kink hydrogen bond length, Supplementary Fig. 21c-d), while the other two subunits remained in the closed state.

Note that bending/kinking of the inner helices in the above simulations sometimes happened on the helical turns following the kink around Met298 (residues 300-306), which was partially responsible for the observed cavity hydration. In this regard, we conducted control simulations with these backbone hydrogen bonds restrained (Supplementary Table 2) to evaluate the effects of the unstable protein structure. Similar to simulations without restraints, conformational transitions of the four subunits happened asymmetrically and we obtained

partially hydrated channels whose average structures were moderately similar to the average structure of the open state channel (Supplementary Fig. 22). Restraining these hydrogen bonds reduced the probability of the conformational transition in enforced hydration simulations (5 out of 8), but for those in which conformational transition happened, the average structures are more similar to the open state channel (reduced RMSD in the boxplot in Supplementary Fig. 22b), as expected. In the mutant simulations, restraining these hydrogen bonds also reduced the probability of conformational transition, as suggested by the decreased cavity hydration level (Supplementary Fig. 22c).

In summary, hydration of the cavity by either mutation or restraining water molecules was able to induce conformational transition from the closed to the open state, highlighting the coupling between cavity hydration level and channel structure. The asymmetrical behaviour of the four subunits implied that cavity hydration itself may not be able to induce the complete conformational transition from the closed to the open state. In the *in vivo* systems, coupling between the TM domain and the calcium binding domain may provide the driving forces for the conformational transition. We also propose that the repulsion between negatively charged residues in the Ala305Glu mutant may also work as a driving force for conformational transitions in our simulations, in addition to cavity hydration.

Supplementary Note 5 - Conformational Transition of MthK Induced by Cavity Dewetting.

Similar to the BK channels, the main structural difference between the open and the closed states was characterized by different helical bending angle due to a kink at the middle of the inner TM helix (Supplementary Fig. 11a-c). We used the backbone hydrogen bonds around the kink (81O-85HN), helical bending angle, and side chain orientation of Phe87 (corresponding to Phe304 in BK) to compare the structures of different states of MthK (Supplementary Fig. 23e). The TM helical bending angles of the open (PDB entry: 3ldc [http://doi.org/10.2210/pdb3LDC/pdb]) and closed states (PDB entry: 6u6d [http://doi.org/10.2210/pdb6U6D/pdb]) are ~45° and ~8° respectively (Supplementary Fig. 23b, e). We applied the abovementioned two non-equilibrium simulation methods to MthK and we obtained conformation whose cavity was dewetted (average water numbers of ~15, compared to ~45 of the open state, Supplementary Fig. 23d) but not sealed by helical intersection (suggested by a helical bending angle of ~35°, Supplementary Fig. 23a, b, e). The cavity dewetting was ascribed to the hydrophobic side chain of Phe87, which occupied

the central cavity in these conformations but pointed to the interfaces between subunits in the open conformation and fluctuated between two states in the closed conformation (Supplementary Fig. 23a, e). The representative non-equilibrium simulations (Supplementary Fig. 23f, Supplementary Fig. 24) indicated conformational transitions during 0.5-1.0 μs. In these simulations, cavity dewetting and Phe87 side chain orientation remained stable after conformational transition (Supplementary Fig. 23f, Supplementary Fig. 24), but the protein structure (see the RMSD value as a function of simulation time) and helical bending angle fluctuated. All of these data implied the significance of cavity dewetting in the gating procedure of potassium channels which employ the "bundle crossing" mechanism, although what we sampled is likely to be an intermediate state, not a stable closed state.

We note that the success rate of the enforced dewetting simulations was low (4 out of 20, ~20%) compared to the BK channel. The waters were not pulled out of the cavity in the failed simulations, probably due to the Glu92 and Glu96, whose negatively charged side chains created a hydrophilic environment (Supplementary Fig. 25). The relatively large helical bending angle of the intermediate states we obtained may also be ascribed to the repulsion between these residues from the four subunits. Lacking of several residues of the inner TM helices in simulations, which were not solved in the crystal structure of the open state but might be favourable for the helical interactions in the closed structure, is another possible reason for not sampled a stable closed state in our non-equilibrium simulations.

Supplementary Note 6 - Lipid-Protein Interactions.

We conducted 10 parallel simulations of the open state (wild type) without applying the dummy atoms, among which 4 replicates showed lipids entering into the cavity. In the trajectories without lipids distributed in the central cavity, lipids were found to locate in the vicinity the subunit interfaces with their polar headgroups pointing toward the central cavity and the cavity remained fully hydrated (Supplementary Fig. 26d, Supplementary Table 1). However, a larger crack at the subunit interface due to occasional protein structural deformation allowed lipid diffusion laterally into the cavity in some simulation replicas, which may modulate cavity hydration level. In 3 simulations replicas, 1 lipid or part of a lipid penetrated into the cavity and slightly decreased hydration levels were found (water number ~50, Supplementary Fig. 26c), whereas in 1 simulation replica, more than 2 lipids occupied the central cavity and resulted in a complete dehydration (water number <5, Supplementary Fig. 26a-b). Whether this is physiological is debatable (see discussion), and we therefore

applied dummy atoms (see Supplementary Methods) to exclude lipids from the cavity so that we can sample the fully hydrated case. We note that, comparison of the above simulations with those using dummy atoms indicated very minor effects of dummy atoms on ion permeation in case of a fully hydrated cavity: (a) Trajectories in which the central cavity did not accommodate lipids revealed an ion current (10.9±1.2 v.s. 14.4±1.2 pA, difference has no statistical significance) and a hydration level (number of water molecules 56±1.0 v.s. 61±0.8, see Supplementary Table 1) very similar to the simulations using dummy atoms. (b) The free energies for potassium and water molecules entering into the cavity based on these trajectories are comparable to those obtained by simulations using dummy atoms (Supplementary Fig. 27).

The lipid-protein interactions in the closed state (wild type) revealed by 12 parallel equilibrium simulations can be divided into 4 categories (Supplementary Fig. 26e-h): (a) 2 lipids entered into the cavity (found in 2 replicas, Supplementary Fig. 26e); (b) part of a lipid entered into the central cavity (found in 6 replicas, Supplementary Fig. 26f); (c) lipids did not enter (found in 3 replicas, Supplementary Fig. 26g); (d) one lipid tail entered into the cavity (found in 1 replica, Supplementary Fig. 26h). In all cases (a, b and c), lipids entered via the crack between subunit interfaces. In categories a-b, lipid entering was always accompanied by a collapse of the tetramer structure, which created a crack large enough to allow lipid entry. Category d, the rarest case, resembles the situation of MthK in Fan et al.'s work[1] to some extent. In their simulations, four lipid tails entered into the cavity from the four subunit interfaces, whereas entering of only one lipid tail was observed in our case. Since the physiological relevance of lipid entering into the cavity found in these simulations is unclear and an extensive sampling of lipid-protein interactions was not the main purpose of this work, we applied dummy atoms to prevent the effects of lipids on the tetramer structure of the channel (i.e., avoid the situations in cases a-b). The rationale of using dummy atoms was also supported by the following results: (a) the above category c (no lipid entering) and the simulations using dummy atoms showed similar hydration levels (20.9 v.s. 23.0, Supplementary Table 1) and average structures (RMSD 0.12 nm); (b) we took the last snapshots of the simulations using dummy atoms and continued the calculations after removing these dummy atoms. Lipids did not enter into the cavity in these simulations. Besides, the simulations revealed very similar hydration level (17.8 v.s. 23.0, Supplementary Table 1), ion current (0.8 v.s. 1.3), average structures (RMSD 0.08 nm), and free energies for

potassium and water entering into the cavity (Supplementary Fig. 27), compared to the simulations using dummy atoms.

Supplementary Methods.

Protocol for MD simulations.

MD simulations were conducted at 310 K or 330 K using an integration time step of 2 fs. Simulation parameters were the same to our previous work[2]. Briefly, the Nose-Hoover algorithm[3,4], were applied to maintain the temperature of the system at the target values with a relaxion time of 1 ps, whereas the pressure was restrained at 1 bar using the semi-isotropic coupling method and the Parrinello-Rahman algorithm[5,6], with a relaxion time of 5 ps and an isothermal compressibility of 4.5×10^{-5}. The Van der Waals interactions were turned off from 1.0 nm to 1.2 nm using the force-switched method[7]. The long-range electrostatic interactions were handled with the Particle Mesh Ewald method[8,9], using a real-space cut-off value of 1.2 nm.

In the simulations, lipids sometimes entered into the central cavity, occluded the permeation pathway and inhibited ion conduction, both in the open and closed states of the BK channel. To prevent this from happening, we used dummy atoms, which had no interactions with the atoms in the system except for weak repulsion with the carbon atoms of the lipid tails, to exclude the lipids from the central cavity (Supplementary Fig. 2). Specifically, 65 dummy atoms were employed with 64 among them arranged on the surface of a sphere, and the last one located at the center. The distances between the atom at the center and each of those on the surface were restrained as harmonic bonds, with a force constant of 50,000 kJ mol^{-1} nm^{-2} and a bond length of 0.9 nm. Weak repulsion between these dummy atoms were assigned so that they were distributed evenly on the sphere surface during MD simulations. The center atom was restrained to the COM of the four Cα atoms of Phe304 using a force constant of 500 kJ mol^{-1} nm^{-2}. Thus, the surface atoms formed a “wall” which prevented lipid tails from entering into the cavity. The repulsion was applied as Lennard-Jones interactions. The σ and ε values of the Lennard-Jones interactions between dummy atoms are 0.36 nm and 0.23 kJ mol^{-1}, while the corresponding values between dummy atoms and lipid tail carbons are 0.62 nm and 0.08 kJ mol^{-1}, respectively.

In non-equilibrium simulations of enforced dehydration/hydration, we employed the chain reaction coordinate (Supplementary Fig. 1) developed by Hub et al.[10,11] to pull waters out of

(or restrain them in) the cavity. The chain reaction coordinate defines a cylinder, cuts it to slices along the axis and uses the ratio of the number of hydrated slices to the total number of slices as the reaction coordinate (ξ) to control the hydration degree of the cylinder. Briefly, a harmonic potential of $V = k(\xi - \xi_0)^2\ /2$ is applied to the system (ξ_0 is the reference point along the reaction coordinate). The force applied to the system is calculated as $F_\varepsilon = -\partial V/\partial\xi = -k(\xi - \xi_0)$, which was then translated to the atoms by $F_j = F_\xi \cdot \partial\xi/\partial r_j$ (j is the atom index). In the chain reaction coordinate, the atoms (waters) are pulled slice by slice to control the hydration degree. In our simulations, we applied the forces on the oxygen atoms of all of the water molecules in the simulation system.

In the enforced dehydration simulations, we defined a cylinder with a radius of 2 nm ranging from the kink to the C-terminal of the inner TM helices to cover the whole central cavity. The number of slices was set so that the value of reaction coordinate (i.e., ratio of the number of hydrated slices to the total number of slices) for the initial structure was ~0.8. The waters were pulled and the reaction coordinate was reduced to 0 in 0.5 μs using a force constant of 10,000 kJ mol^{-1} nm^{-2} and a pulling rate of 1.6×10^{-6} ps^{-1}. The cavity was then restrained to be empty (i.e., reaction coordinate value of 0) using a force constant of 50,000 kJ mol^{-1} nm^{-2} for another 0.5 μs to relax the system, followed by 0.5 μs simulations without any restraints to equilibrate the structure of the protein. In the enforced hydration simulations, the simulations were started from the closed cryo-EM structure with water molecules restrained in the central cavity. Specifically, a cylinder with a radius of 0.5 nm and a smaller ζ value of 0.6 (which means 60% of a slice is occupied if one water molecule entered the slice, in the enforce dehydration simulations the default value of 0.75 was used) were used to restrain the water molecules with a force constant of 50,000 kJ mol^{-1} nm^{-2} for 0.5 μs simulations. The systems were then equilibrated for another 0.5 μs without restraints.

In the non-equilibrium simulations using dummy atoms. The dummy atom was restrained to the COM of backbone atoms of the kink residues (specifically, the backbone oxygen of Met298 and Ile299 and the backbone nitrogen of Gly302 and Leu303) using a force constant of 1000 kJ mol^{-1} nm^{-2}. The repulsion between dummy atoms and water oxygens were applied as Lennard-Jones interactions with the σ and ε values set to be 0.62 nm and 0.08 kJ mol^{-1}, respectively.

Data Analyses.

Ion permeability. The ion currents across the channel, as well as the free energy profiles of potassium ions/water molecules entering into the cavity, were used to characterize the ion permeability of different states and mutants of the channel. The calculation method was the same to our previous work[2]. For the current, the permeation events during MD simulations were counted (a potassium ion translocated from the S_c binding site to the S_0 binding site and then left the S_0 binding site was counted as one permeation event) and the ion current was calculated by dividing the permeated charges by the simulation time. The Fortran code used for this calculation can be found in our previous work[2,12].

For the free energy profile, we first calculated the number distributions of potassium ions and waters along the permeation pathway and generated the free energies based on their distributions. To calculate the number distributions, we superimposed the snapshots based on the selectivity filter (i.e., the heavy atoms of Thr276 and the backbone atoms of Val277, Gly278, and Phe279), and then counted the number of the potassium ions/waters in a cylinder with a radius of 1.0 nm (i.e., approximately the size of central cavity, a smaller radius of 0.275 nm was used for counting water molecules in the selectivity filter region to avoid counting those which bind behind the selectivity filter), whose axis was parallel to the permeation pathway and centered on the center of mass (COM) of Thr276 side chains. The number distributions and free energy profiles were then calculated.

Number of water molecules and potassium ions in the cavity. We used the number of water molecules in the central cavity to measure the hydration levels of different states and mutants of the channel. We first defined the central cavity (Supplementary Fig. 3) as a series of slices with a width of 0.2 nm, which were centered at different positions along z direction. The center of each slice was calculated as the center of mass (COM) of the backbone atoms of the inner TM helices located in the same positions along the z direction, while the radius was defined as the average distance between the COM of each TM helices to the center of the slice, as shown in Supplementary Fig. 3. The number of water molecules in each slice was counted and the sum of the numbers over all slices was considered as total water numbers in the cavity.

For equilibrium simulations, the whole production trajectories of all simulation replicas were used to calculate the water numbers, while for non-equilibrium simulations, the last 0.25 μs

trajectories were used. For MthK, as we did not preform equilibrium simulations, water numbers of the first 5 ns of the non-equilibrium simulations were calculated and considered as the hydration level of the open state.

The number of potassium ions in the cavity were calculated by the same method. However, we only considered the potassium ions which were located in the cavity but did not bind at the S_c site when calculating their solvation degree, as S_c-bound potassium ions were coordinated by the oxygen atoms from the protein instead of the water molecules. Potassium ions which located in the cavity but within 0.25 nm of the centre of mass of the Oγ atoms of Thr276 residues along the z axis were considered to be bound at the S_c site. Water molecules whose oxygen atoms were within 0.35 nm and 0.61 nm of potassium ions were considered to be the first and second solvation shells. These cutoff values were determined based on the first two local minima of the potassium ion-water oxygen radial distribution obtained based on a simulation of KCl solution with a concentration of 1 M.

Characterization of protein conformation. We employed three different data to characterize the protein conformation, particularly the conformation of the inner TM helices. These data are backbone "hydrogen bonds" around the kink region and around Pro309, distances between Cα atoms of Phe304 from the opposite subunits, and the Phe304 side chain orientation.

A kink of the inner TM helices formed due to Gly302 was considered as a key feature of the open state of the BK channel (Fig. 2a). The distance between Ile297 backbone oxygen and the Ile301 hydrogen of the amino group (297O-301HN), and the corresponding distance between Met298 and Gly302 (298O-302HN) were calculated and their distributions were used to characterize the kink conformation, as shown in Fig. 2b. These distances were referred to as "hydrogen bonds", although they were not always formed hydrogen bonds in different states. Similarly, other hydrogen bond lengths and their distributions were employed to describe the conformation of the other part of the inner TM helices (specifically, 301O-305HN, 302O-306HN, 303O-307HN, 304O-308HN, 305O-309N, 306O-310HN, see Supplementary Fig. 11, note that the distance between backbone oxygen and backbone nitrogen were used for the "hydrogen bonds" between Ala305 and Pro309, as the Pro309 does not has an amino group hydrogen).

The hydrophobic Phe304 side chain was inferred to be responsible for cavity dewetting in the closed state. We described its orientation/conformation by an angle between two vectors: the first vector was defined from the position of its Cα atom to the COM of its side chain, while the second vector was defined from the Cα atom to the COM of Cα atoms of the four subunits, as shown in Fig. 2c.

Structural comparison. Structures averaged over the MD trajectories were calculated and the RMSD values between these average structures were used to compare protein conformations in different simulations. In equilibrium simulations, the whole production trajectories of all simulation replicas were subjected to average structure calculation. In non-equilibrium simulations, the last 0.25 μs trajectories were used to calculate the average structure. Note that in the case of non-equilibrium simulations, average structure of each replica and the average structure of all replicas were calculated separately. The pore helix, the selectivity filter and the inner TM helix of the protein were used to calculate the RMSD values. RMSD values of the average structure of each replica in non-equilibrium simulations, and corresponding value of the average structure of all simulation replicas, were calculated separately relative to the average structure of the equilibrium simulations. For the representative non-equilibrium simulations, RMSD of the snapshots from the trajectories were calculated using the open and closed average structures in equilibrium simulations as the references to characterize the protein conformational change.

Supplementary Table 1. Summary of MD simulations of BK channel in this work[a].

	State/ Mutations of init. Conf.	Temp. (K)	Time (μs) × # of replicas	Curr. (pA)	# of Wat. in cavity	K^+ Energy Barrier (k_BT)	Wat. Energy Barrier (k_BT)	RMSD[b] (nm)	RMSD[c] (nm)
Equilibrium simulations, 300 mV, $[K^+]$ = 1 M, No restraints applied	open/WT	310	0.5×10	14.4±1.2	61.0±0.8	2.5	0.8	-	-
	open/A305E		0.5×10	15.0±1.6	58.9±1.8	no barrier	1.0	-	-
	open/A305V		0.5×5	6.8±1.4	39.9±7.5	3.2	1.3	-	-
	open/A305L		0.5×5	0.3±0.1	18.1±1.6	9.2	3.0	-	-
	closed/WT	310	0.5×10	1.3±0.4	23.0±0.9	7.1	2.1	-	-
	closed/A305E		0.5×10	18.7±1.6	57.5±1.6	0.2	1.0	0.21±0.01	0.19
	closed/A305V		0.5×5	1.7±0.8	27.0±2.0	6.3	2.1	-	-
	closed/A305L		0.5×5	1.7±1.1	22.8±6.0	6.4	2.3	-	-
	closed/F304A		0.5×10	11.0±1.1	54.4±1.2	3.7	1.0	-	-
	closed/I308A		0.5×10	7.4±1.4	43.9±2.6	3.4	1.1	-	-
	closed/F304A-I308A		0.5×10	9.5±1.5	67.5±2.4	2.8	1.0	-	-
	closed/G302A	310	0.5×10	0.3±0.1	15.9±1.2	infinite	2.5	-	-
	closed/P309A		0.5×10[d]	0.6±0.4	6.6±2.3	8.8	3.9	-	-
	closed/A312V	310	0.5×5	1.6±0.9	26.6±2.3	5.1	1.9	-	-
Equilibrium simulations, 300 mV, $[K^+]$ = 1 M, restraints of hydrogen bonds	closed/WT state1	310	0.5×5	1.2±1.0	15.7±3.2	6.1	2.3	-	-
	closed/WT state2		0.5×5	0.4±0.2	21.8±0.9	7.7	2.4	-	-
Equilibrium simulations[e], 150 mV, $[K^+]$ = 1M,	open/WT	310	0.5×8	7.9±1.2	60.7±1.6	2.5	0.7	-	-
	closed/WT	310	0.5×8	0.5±0.3	21.2±1.5	5.1	2.1	-	-

No restraints applied									
Equilibrium simulations, 300 mV, $[K^+]$ = 0.15 M, No restraints applied	open/WT	310	0.5×8	11.0±0.7	62.2±1.3	1.2	0.7	-	-
	open/A305V	310	0.5×8	5.4±0.7	49.9±3.4	1.6	1.1	-	-
	open/A305L	310	0.5×8	1.0±0.7	20.5±1.8	4.2	2.8	-	-
	closed/WT	310	0.5×8	0.6±0.3	24.1±1.1	4.2	2.1	-	-
	closed/A305E	310	0.5×12	16.8±1.6	65.2±2.0	no barrier	0.8	-	-
	closed/F304A	310	0.5×12	3.2±0.6	53.1±0.9	2.3	1.1	-	-
	closed/I308A	310	0.5×12	3.1±0.6	45.5±3.3	2.5	1.1	-	-
	closed/F304A-I308A	310	0.5×12	3.6±0.4	71.1±1.4	2.0	0.9	-	-
Equilibrium simulations, 150 mV, $[K^+]$ = 0.15 M, No restraints applied	open/WT	310	0.5×8	4.6±0.3	60.6±1.3	1.2	0.7	-	-
	open/A305V	310	0.5×8	2.0±0.3	46.6±3.6	1.8	1.1	-	-
	open/A305L	310	0.5×8	0.2±0.1	17.3±0.6	7.2	3.4	-	-
	closed/WT	310	0.5×8	0.5±0.2	24.1±1.2	4.4	2.0	-	-
	closed/A305E	310	0.5×8	5.1±0.8	60.9±1.9	no barrier	0.8	-	-
	closed/F304A	310	0.5×8	1.5±0.3	53.5±1.7	3.0	1.2	-	-
	closed/I308A	310	0.5×8	0.9±0.2	43.2±2.8	2.9	1.2	-	-
	closed/F304A-I308A	310	0.5×8	1.7±0.2	71.4±3.3	2.5	1.0	-	-
Equilibrium simulations, 300 mV, $[K^+]$ = 1 M, dummy atoms not applied, backbone of inner helices restrained	closed/WT	310	0.5×10	13.3±1.8	80.6±0.5	2.4	0.7	-	-

Equilibrium simulations, 300 mV, $[K^+]$ = 1 M, dummy atoms not applied, no restraints applied	open/WT[f]	310	0.5×10	10.9±1.2	55.5±1.0	2.8	0.8	-	-
	closed/WT[g]	310	0.5×10	0.8±0.3	17.8±1.4	6.5	2.3	-	-
Equilibrium simulations, no voltage, $[K^+]$ = 1M, dummy atoms not applied, no restraints applied	closed/WT[h]	310	12 replicas, Sim. Time of 0.2, 0.2, 0.35, 0.32, 0.38, 0.43, 0.48, 0.44, 0.46, 0.34, 0.47, 0.50	-	20.9±3.8	-	-	-	-
Non-equilibrium, enforced dehydration, no voltage	open/WT	310	1.5×17[i]	-	13.8±2.3	-	-	0.24±0.01	0.12
	open/G302A		1.5×10	-	8.3±1.8	-	-	0.20±0.01	0.10
	open/P309A		1.5×20[j]	-	18.3±2.3	-	-	0.28±0.01	0.20
Non-equilibrium with dummy atoms, no voltage	open/WT	330[k]	10 replicas, Sim. Time of 2.498, 2.488, 2.0, 2.011, 1.5, 4.5, 0.6, 2.498, 1.073, and 1.073	-	18.7±2.1	-	-	0.24±0.01	0.12
Non-equilibrium with dummy atoms and hydrogen bond restraints, no voltage	open/WT		7 replicas, Sim. Time of 0.893, 0. 901, 1.729, 1.9, 1.688, 1.95, 1.742	-	17.2±3.7	-	-	0.22±0.01	0.10

Equilibrium simulations, no voltage, $[K^+]$ = 1 M	closed/A305E	310k	0.2×5	-	64.1±3.0	-	-	0.22±0.01	0.17
Equilibrium simulations, No voltage, $[K^+]$ = 1 M, hydrogen bond restraints	closed/A305E	310	0.2×10	-	53.5±3.2	-	-	0.27±0.01	0.20
Non-equilibrium, enforced hydration, no voltage	closed/WT	310	1.0×12	-	46.6±3.8	-	-	0.33±0.02	0.23
Non-equilibrium, enforced hydration, no voltage, hydrogen bond restraints	closed/WT[l]	310	1.0×8	-	49.1±2.3	-	-	0.27±0.03	0.20

a, The errors shown in this table are standard errors of the mean (s.e.m.).

b-c, RMSD of the average structures in non-equilibrium simulations relative to the average structure of the equilibrium simulations of the other state (i.e., if the simulations started from the closed state, the RMSD values were relative to the open state, and *vice verse*).

b, Average structure was calculated for each replica respectively and the RMSD value was calculated for each of these average structures. The average values and the standard errors of the mean (s.e.m.) were presented.

c, Structure was averaged over all replicas and the RMSD values of the average structures were shown.

d, The tetramer arrangement collapsed in some of the Pro309Ala simulations (5 out of 10), and the data shown here were those in which the tetramer structure was maintained.

e, The energy barriers of potassium entering into the cavity were systematically smaller in simulations of [K] = 0.15 M than those of [K] = 1 M, due to the relatively low concentration of ions in the bulk region. The shapes of the profiles were not changed, see Supplementary Fig. 9.

f, Lipid distributions in the cavity were found in 4 replicas. Data of these 4 replicas were based on parts of the trajectories in which lipids were not found in the cavity. In these simulations, the dummy atoms were not applied from beginning of the simulations.

g, Snapshots at the end of the simulations using dummy atoms (1 M/0.3 V) were extracted and used as the initial conformations for these simulations after removal of the dummy atoms.

h, Cryo-EM structure of the closed state channel was used as the initial conformation for these simulations. Dummy atoms were not applied from beginning of these simulations. Lipid distributions were not found in 3 simulation replicas. Numbers of water molecules were calculated based on these 3 simulations. The last 50 ns simulations were used in the analyses.

i, 13 out of 17 simulations successfully induced conformational changes. Data of those 13 simulations in which conformational transition happened were presented.

j, 19 out of 20 simulations successfully induced conformational changes. Data shown here were for those 19 simulations.

k, a higher temperature was used for enhanced sampling.

l, 5 out of 8 simulations successfully induced conformational changes. Data shown here were form those 5 simulations.

Supplementary Table 2. Hydrogen bond restraints used in the MD simulations of BK in this work.

**equilibrium simulations,** closed state, **restraining inner TM helices around Pro309** state 1			**equilibrium simulations,** closed state, **restraining inner TM helices around Pro309** state 2			**non-equilibrium simulations,** started from open state, **restraining inner TM helices around Val294**		
H-bond	Dist. (nm)	force constant (kJ mol^{-1} nm^{-2})	H-bond	Dist. (nm)	force constant (kJ mol^{-1} nm^{-2})	H-bond	Dist. (nm)	force constant (kJ mol^{-1} nm^{-2})
303O-307HN	0.20	2500	303O-307HN	0.26	2500	293O-297HN	0.2	2000
304O-308HN	0.21		304O-308HN	0.41		294O-298HN		
305O-309HN	0.45		305O-309HN	0.59		295O-299HN		
306O-310HN	0.29		306O-310HN	0.22		296O-300HN		
**equilibrium &non-equilibrium simulations**, started from the closed state, **restraining inner TM helices following the kink region,** for simulations to induce conformational transition from the closed to the open state								
H-bond	Dist. (nm)	force constance (kJ mol^{-1} nm^{-2})						
300O-304HN	0.20	2500						
301O-305HN	0.21							
302O-306HN	0.21							
303O-307HN	0.20							
304O-308HN	0.23							
305O-309HN	0.45							
306O-310HN	0.29							

Supplementary Table 3. Summary of MD simulations of MthK in this work[a].

	State/ Mutations of init. Conf.	Temp. (K)	Time (μs) × # of replicas	Curr. (pA)	# of Wat. in cavity	K^+ Energy Barrier (k_BT)	Wat. Energy Barrier (k_BT)	RMSD[b] (nm)	RMSD[c] (nm)
Non-equilibrium, enforced dehydration	open/WT	310	1.5×20[d]	-	14.4±1.9	-	-	0.41±0.01	0.40
Non-equilibrium with dummy atoms	open/WT	310	8 replicas, Sim. Time of 3.827, 2.788, 5.463, 4.969, 6.593, 5.332, 6.401, 4.240	-	16.1±3.1	-	-	0.42±0.01	0.41

a, The errors shown in this table are standard errors of the mean (s.e.m.).

b-c, RMSD of the structures in non-equilibrium simulations relative to the cryo-EM structure of the closed state (PDB entry: 6u6d [http://doi.org/10.2210/pdb6U6D/pdb]).

b, Average structure was calculated for each replica respectively and the RMSD value was calculated for each of these average structures. The average values and the standard errors of the mean (s.e.m.) were presented.

c, Structure was averaged over all replicas and the RMSD values of the average structures were shown.

d, 4 out of 20 simulations successfully induced conformational changes. Data shown here are for those 4 simulations in which conformational transition happened.

Supplementary Table 4. Solvation degree of potassium ions in bulk and in the central cavity of potassium channel. Number of water molecules with in the first shell (distances between water oxygens and ions <0.35 nm) and the first two (<0.61 nm) shells of potassium ions are shown. The errors are standard error of the mean (s.e.m.). The cutoff values for calculations were determined based on the first two local minimums of the potassium-water oxygen radial distribution function in bulk in Supplementary Fig. 6.

**Simulation System**		**1st shell**	**1st&2nd shell**
**bulk**		6.6±0.0*	31.0±0.0
**open**	**WT**	6.5±0.0	25.6±0.1
	**A305E**	5.5±0.1	22.0±0.3
	**A305V**	6.3±0.1	22.9±0.8
	**A305L**	6.0±0.2	20.0±0.7
**closed**	**WT**	6.2±0.1	19.1±0.4
	**A305E**	6.0±0.1	22.8±0.3
	**A305V**	6.3±0.1	20.4±0.4
	**A305L**	6.2±0.1	18.5±1.1
	**F304A**	6.5±0.0	24.8±0.2
	**I308A**	6.3±0.0	22.1±0.3
	**F304A-I308A**	6.5±0.0	25.2±0.2

*** a s.e.m. of 0 means the s.e.m. < 0.1**

Supplementary Figure 1. A model illustrating the chain reaction coordinate.

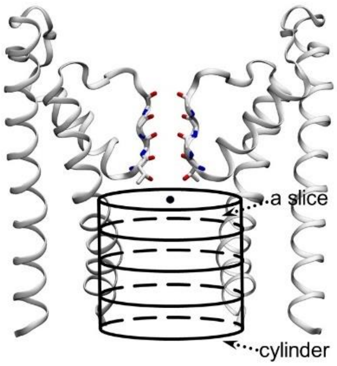

Supplementary Figure 2. Dummy atoms are used to prevent lipids from entering into the central cavity. Protein backbone are shown in white ribbons, while the dummy atoms are shown in space filling model in cyan. Left: side view; Right: top view.

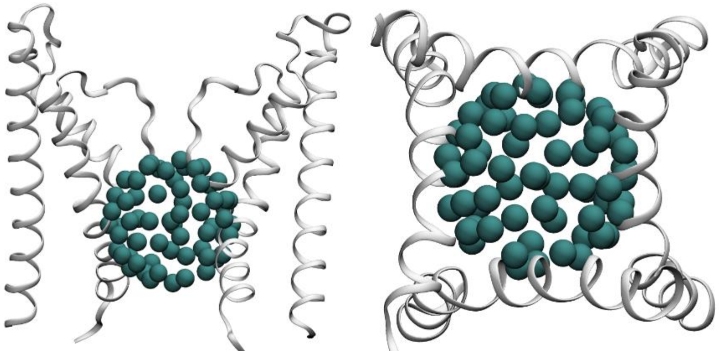

Supplementary Figure 3. Definition of the central cavity space, which is used to calculate the number of waters. Left: the space is defined by a serious of slices with different radius; Right: the center of a slice is defined by the COM of the corresponding backbone atoms from the four subunits, while the radius of the slice is defined as the average of the distances between the slice center and the COM of each subunit.

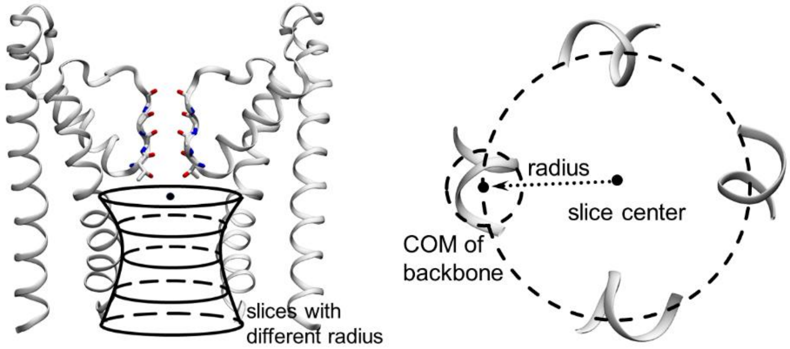

Supplementary Figure 4. Free energy profiles of potassium ions and water molecules across the permeation pathway. (a) Free energy profiles of potassium ions. (b) Free energy profiles of water molecules. Results are shown for the simulations of the wild type and mutants of both the open and closed states. "dbl. Mut." refers to the F304A/I308A double mutant. Correspondence between the x axis and the positions along the permeation pathway are guided by gray dashed lines, which roughly label the positions of I308, F304, and the potassium binding sites of S_c-S_0. Residues F300 and I301 are also shown as they may responsible for the energy barrier after F304. Residues G280 and F283 are shown as they may explain the barrier for potassium leaving binding site S_0. The waters rarely entered into the selectivity filter and their free energies at some positions are so high that they are cut by the y axis.

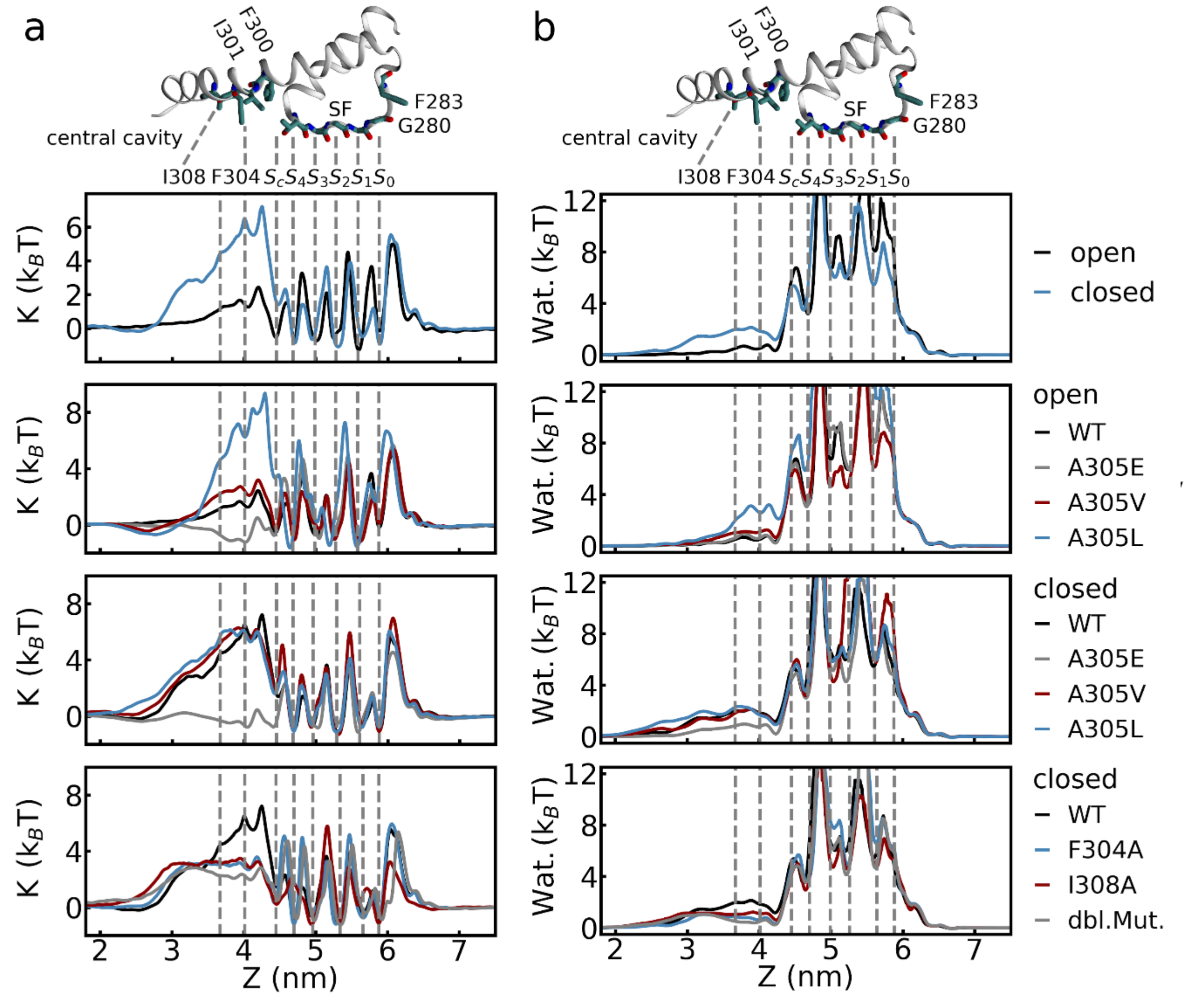

Supplementary Figure 5. Effects of mutations on the conformation of the aBK channel. Average structures of the wild type and mutant simulations were calculated for comparison. (a) RMSD values of the average structures of the mutants relative to the average structures of the open and closed states of the wild type. The A305E mutation of the closed state was highlighted by orange, as it induced a conformational transition form the closed to the open state. (b) Distributions of the hydrogen bonds at the kink for the wild type and mutants. Distributions of the open and closed states of the wild type are shown in black and red in all panels for comparison. (c) Alignment of the average structures of the mutants and those of the open and closed states of the wild type. The A305E mutant was aligned to both the open and closed states to show its conformational transition. Structures of the wild type channel and the mutants are shown in white and cyan.

a

RMSD (nm)

	open A305E	open A305V	open A305L
open/WT	0.07	0.11	0.19
closed/WT	0.32	0.26	0.22

	closed A305E	closed A305V	closed A305L
open/WT	0.19	0.38	0.43
closed/WT	0.22	0.12	0.15

	closed I308A	closed F304A	closed dbl.Mut
open/WT	0.38	0.33	0.35
closed/WT	0.13	0.10	0.11

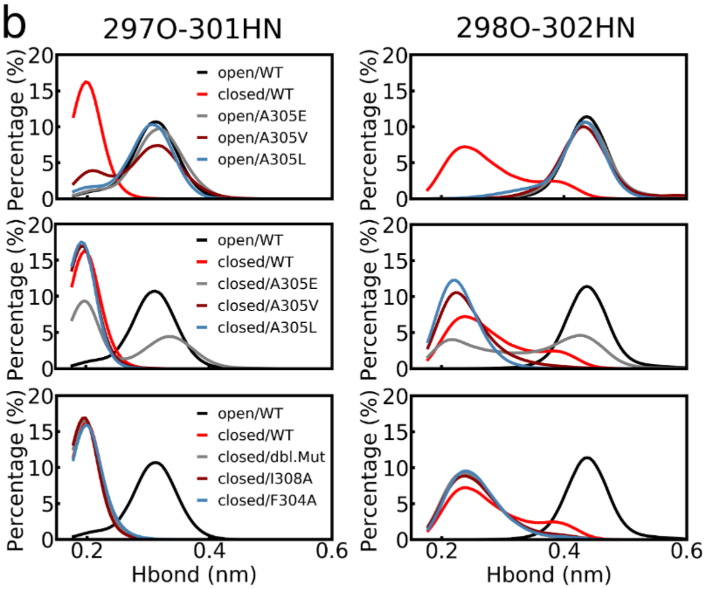

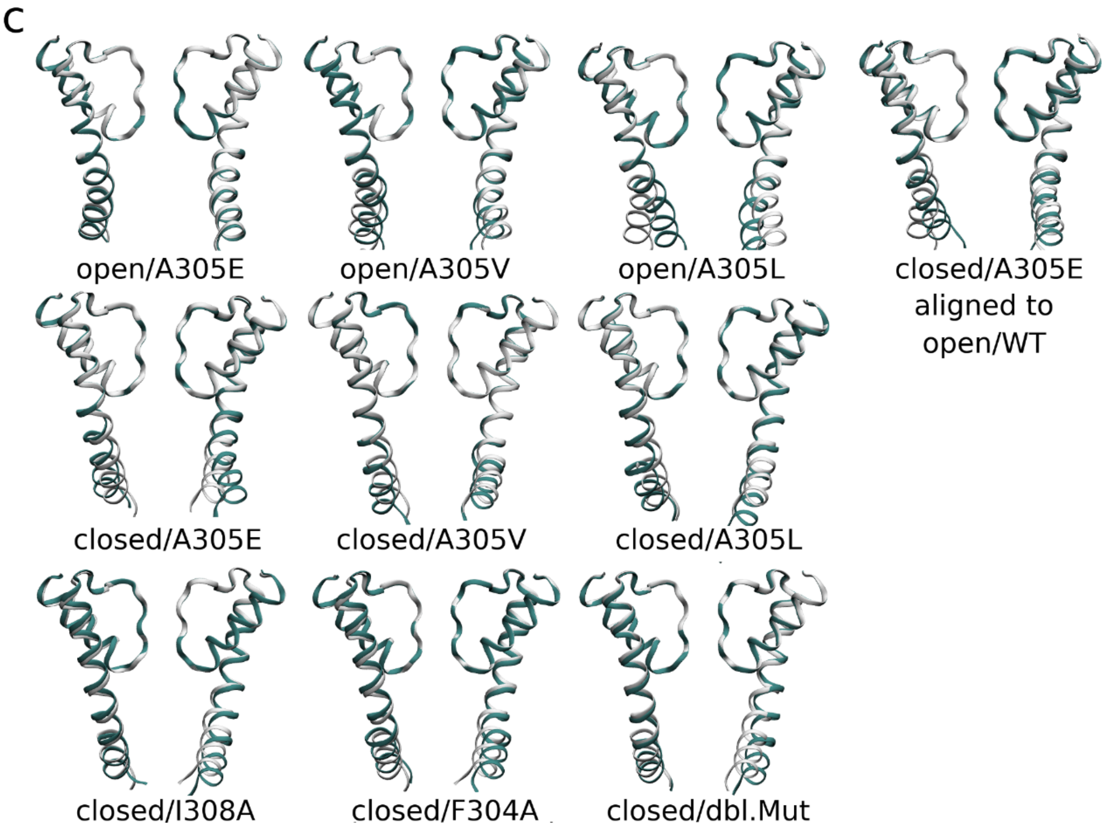

Supplementary Figure 6. Solvation degree of potassium ions in the central cavity. (a) Radial distribution function (RDF) of oxygen atoms of the water molecules around potassium ions in bulk. (b) Distributions of the numbers of water molecules within the first two solvation shells of potassium ions (i.e., distance between oxygen and ion <0.61 nm) in the central cavities. Results of the mutants are compared to the open and closed states of the wild type channel.

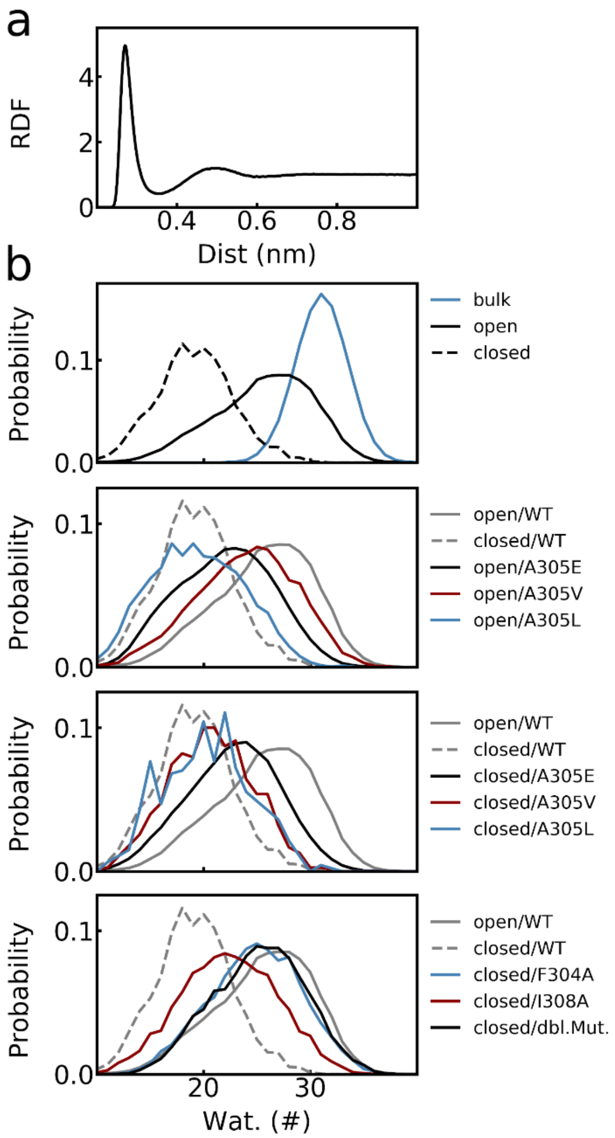

Supplementary Figure 7. Water densities in the central cavity in MD simulations of the BK channels. Water density was defined as the number density of oxygen atoms. The density was normalized relative to the density in the bulk.

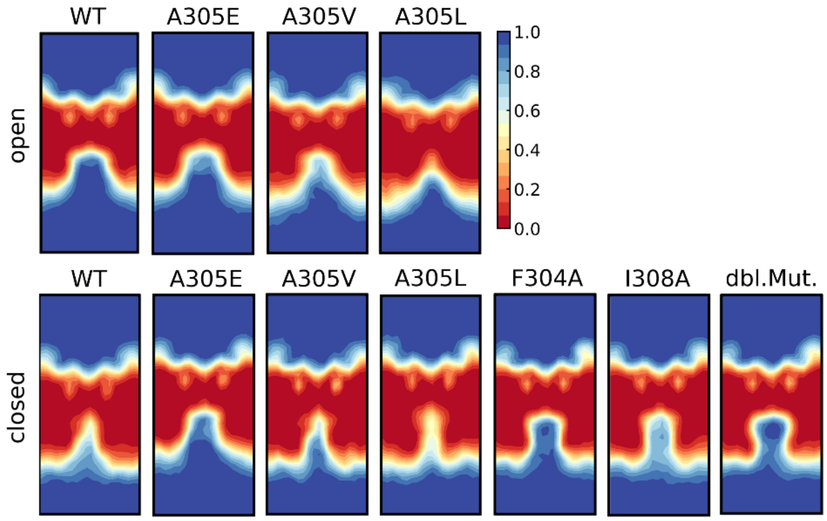

Supplementary Figure 8. Effects of transmembrane voltage and potassium concentration on ion conduction of BK. (a) Ion currents and numbers of water molecules in the cavity of the open and closed states of the WT channel. We tested four situations: 1 M/0.3 V, 1 M/0.15 V, 0.15 M/0.3 V and 0.15 M/0.15 V. The corresponding values of the mutants and the WT channels under (b) 0.15 M/0.3 V and (c) 0.15 M/0.15 V. The data are presented as boxplot. The centre line, box limits, and whiskers represent the median, upper and lower quartiles, and 1.5 × interquartile range. The data points are in gray dots, while the averages and errors (standard error of the mean, s.e.m.) are in red. “dbl Mut.” stands for the F304A/I308A double mutant. (d) Correlations between currents and numbers of water molecules in simulations under 0.15 M/0.3 V and 0.15 M/0.15 V. (e) Currents, (f) numbers of water molecules and (g) numbers of K^+ in the cavity under different voltages and ion concentrations were compared. We compared the results under 0.15 M/0.3 V and 1 M/0.3 V to illustrate the impact of ion concentration, and the results under 0.15 M/0.3 V and 0.15 M/0.15 V to show the impact of voltage. Grey dashed lines are shown to guide the comparison. Note that numbers of K^+ of A305E are not shown for an easier comparison as these numbers are outliers. The errors in panels d-g are standard error of the mean. n=10, n=8, n=8 and n=8 independent simulations were conducted for both the open and closed wild type channel at 1 M/0.3 V, 0.15 M/0.3 V, 1 M/0.15 V and 0.15 M/0.15 V. n=8, n=8, n=12, n=12, n=12 and n=12 independent simulations were performed for the open/A305V, open/A305L, closed/A305E, closed/I308A, closed/F304A and closed/dbl.Mut channels at 0.15 M/0.3 V, whereas n=8 independent simulations were employed for all of the mutants at 0.15 M/0.15 V. This figure is based on simulations without any restraints on the proteins.

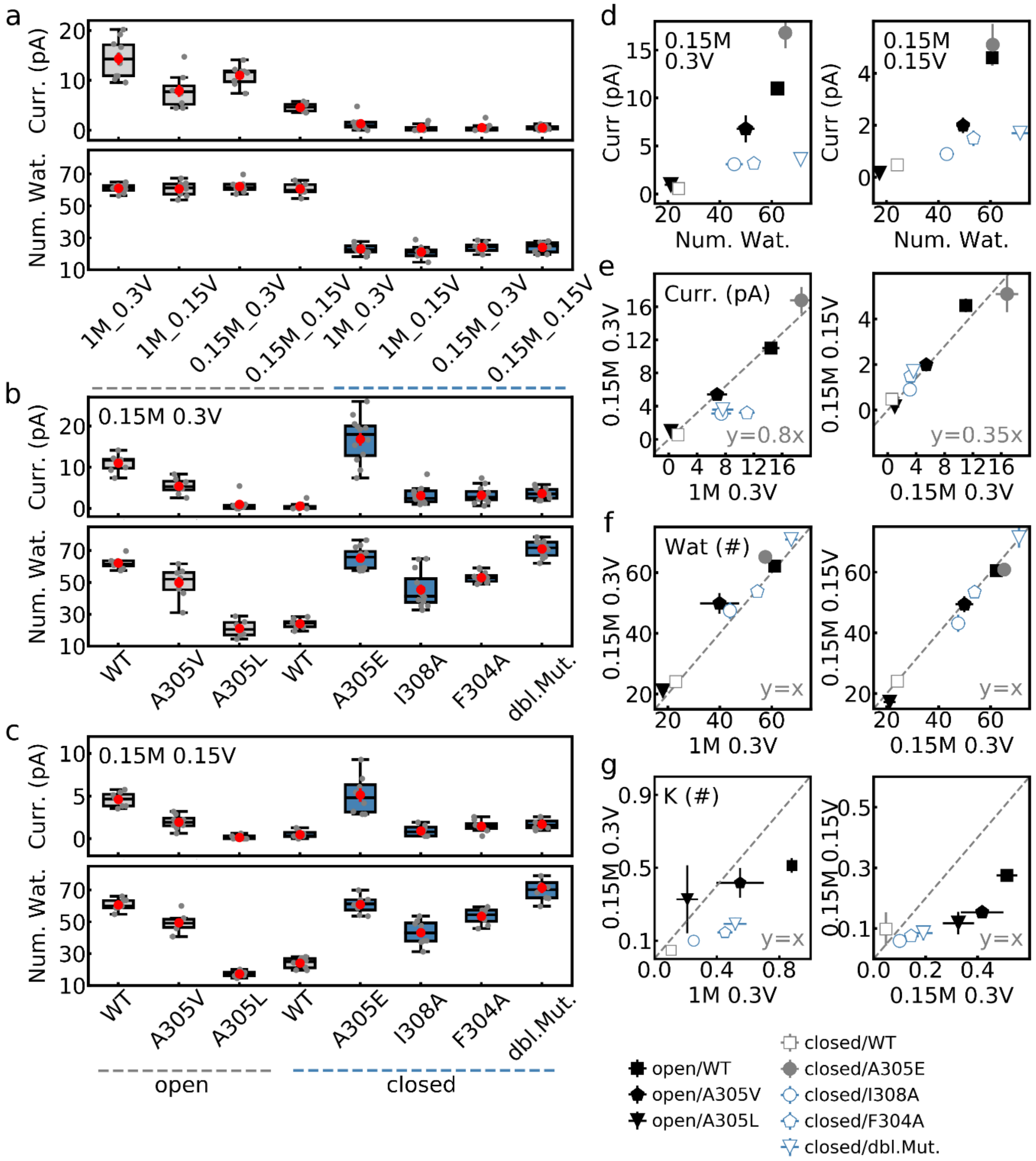
a
Curr. (pA)
Num. Wat.
1M_0.3V
1M_0.15V
0.15M_0.3V
0.15M_0.15V
b
0.15M 0.3V
WT
A305V
A305L
A305E
I308A
F304A
dbl.Mut.
c
0.15M 0.15V
open
closed
d
0.15M
0.3V
0.15V
Curr (pA)
e
Curr. (pA)
1M 0.3V
0.15M 0.3V
0.15M 0.15V
y=0.8x
y=0.35x
f
Wat (#)
y=x
g
K (#)
open/WT
open/A305V
open/A305L
closed/WT
closed/A305E
closed/I308A
closed/F304A
closed/dbl.Mut.

Supplementary Figure 9. Comparison of the free energy profiles in simulations using different potassium concentrations. Results of the potassium ions and water molecules in the simulations using 1 M/0.3 V and 0.15 M/0.3 V are shown for the open and closed states of the wild type channel.

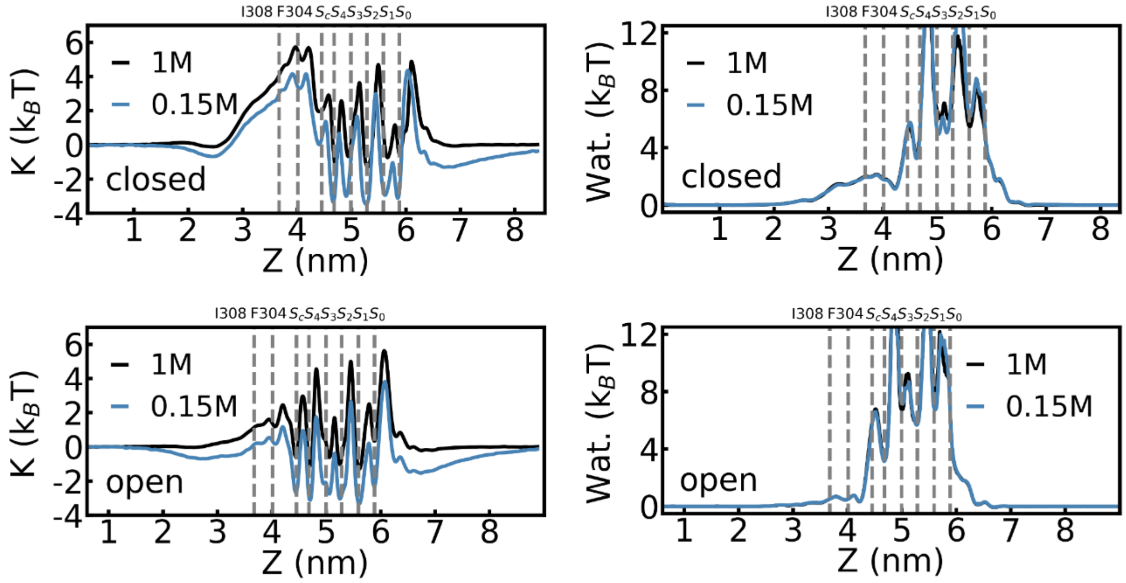

Supplementary Figure 10. Fluctuations of the cavity hydration levels in the simulations of the aBK channel. (a-b) The relative standard deviation (RSD, ratio of standard deviation to the mean of the numbers of water molecules in the cavity) is used to measure the fluctuations of the cavity hydration level. (a) RSD values for the simulations at 1 M/0.3 V. The data are presented as boxplot. The centre line, box limits, and whiskers represent the median, upper and lower quartiles, and 1.5 × interquartile range. The data points of simulation replicas are shown in gray dots, while the averages and errors (standard error of the mean, s.e.m.) are shown in red. "dbl Mut." stands for the F304A/I308A double mutant. (b) RSD values as function of the cavity hydration levels (numbers of water molecules in the cavity). Results of open/A305V, open/A305L, closed/A305V, and closed/A305L in panels a-b include 5 simulation replicas, while results of the other systems include 10 simulation replicas. (c-f) A representative simulation of the closed state of the wild type channel is used to show the fluctuation of the cavity hydration in the case of low hydration level. (c) Numbers of water molecules in the central cavity as function of simulation time. (d) Positions of three water molecules (z coordinates of the oxygen atoms) as a function of simulation time showing the procedure of entering and leaving the cavity of the channel. The position range of the cavity is labeled by a gray shadow. (e) Snapshots showing different cavity hydration levels at different time points of the trajectory. These time points are labeled by numbers 1-4 in panel c. (f) Trajectories of the three waters molecules in panel d in the cavity. Waters at different time points are shown together by different colors.

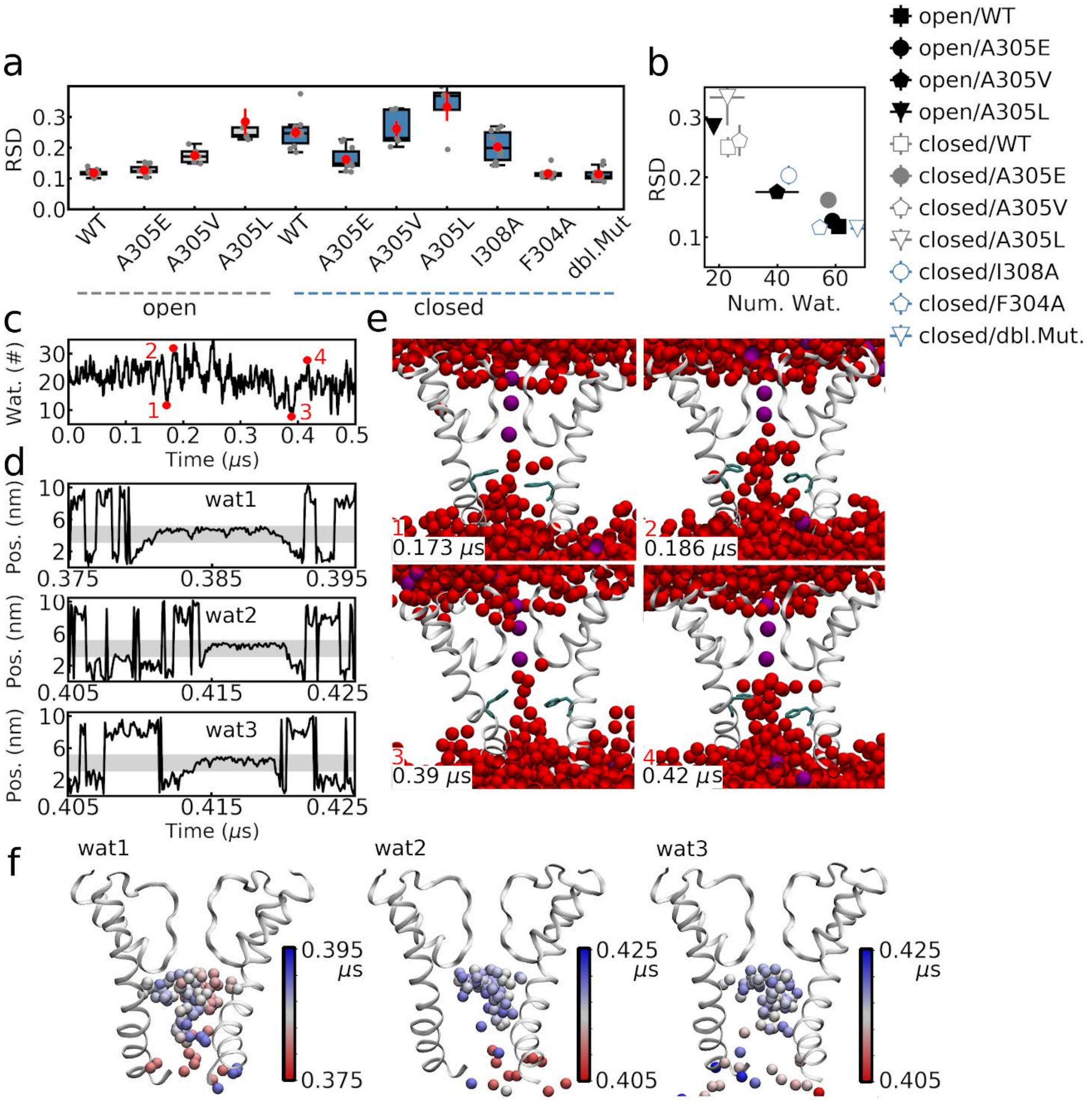
a
RSD
0.0
0.1
0.2
0.3
WT
A305E
A305V
A305L
WT
A305E
A305V
A305L
I308A
F304A
dbl.Mut
open
closed
b
RSD
0.1
0.2
0.3
20
40
60
Num. Wat.
open/WT
open/A305E
open/A305V
open/A305L
closed/WT
closed/A305E
closed/A305V
closed/A305L
closed/I308A
closed/F304A
closed/dbl.Mut.
c
Wat. (#)
10
20
30
1
2
3
4
0.0
0.1
0.2
0.3
0.4
0.5
Time (μs)
d
Pos. (nm)
wat1
0.375
0.385
0.395
wat2
0.405
0.415
0.425
wat3
Time (μs)
e
1
0.173 μs
2
0.186 μs
3
0.39 μs
4
0.42 μs
f
wat1
wat2
wat3
0.395 μs
0.375
0.425 μs
0.405

Supplementary Figure 11. The closed state of BK channel showed two conformations in equilibrium simulations. 2-D distributions for the lengths of the hydrogen bonds of the inner TM helices are used to describe the correlations between these hydrogen bonds, which clearly showed two states. Positions of the involved residues and hydrogen bonds at the inner TM helix are also labelled in a snapshot in the left panel.

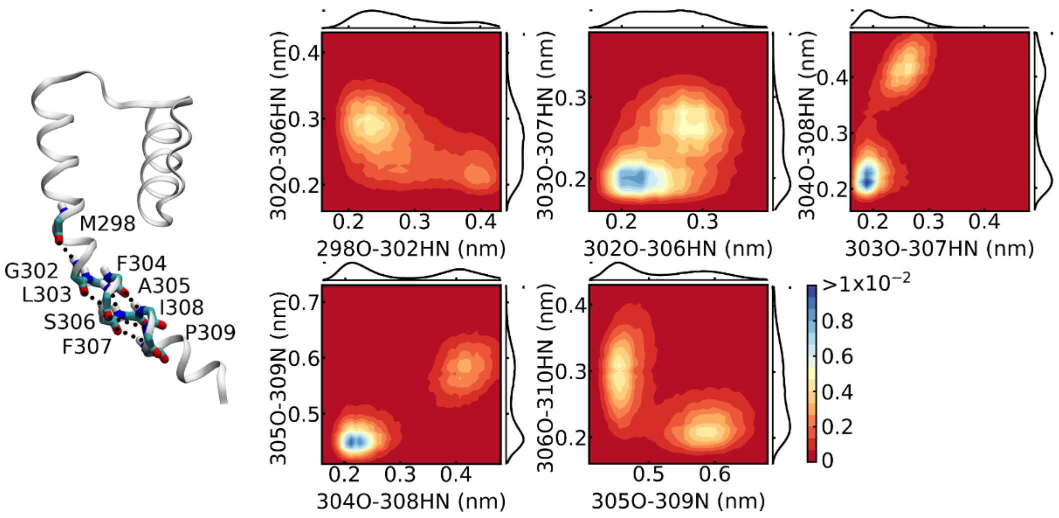

Supplementary Figure 12. The two states of the closed BK channel showed similar hydration degree and ion permeability. (a) Comparison of the conformations of the two states. The average structures of the MD simulations restraining the inner TM helices to two states are superimposed (State 1: white; State 2: cyan). (b) Ion currents and numbers of waters in the cavity for the simulations of two states. The results are shown in boxplot. The centre line, box limits, and whiskers represent the median, upper and lower quartiles, and 1.5 × interquartile range. The data points are shown in gray dots, while the averages and standard errors of the means are shown in red. n=10, n=5 and n=5 independent simulation replicas were conducted for the systems without restraints, state 1 and state 2, respectively. (c) Free energy profiles of potassium ions and waters entering the cavity. (d) Distributions of F304 side chain orientations. Results of the two states are compared to the simulations without restraints (labelled as "State 1", "State 2", and "no rest.") in panels b-d.

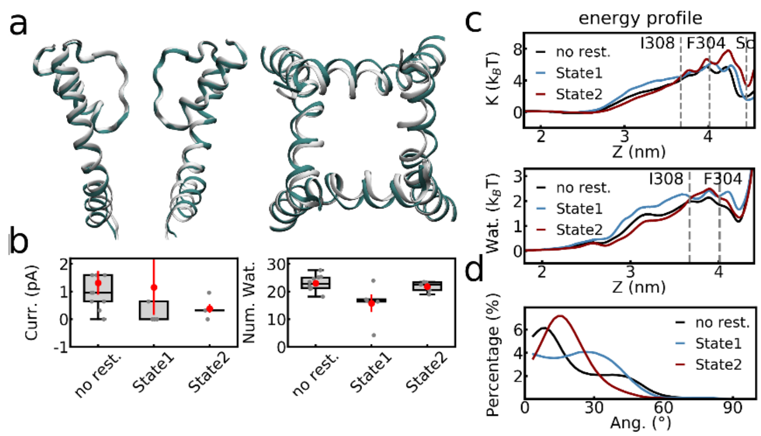

Supplementary Figure 13. Conformation of the inner TM helices of the closed structure obtained from enforced dehydration. Length distributions of the hydrogen bonds of the inner TM helices are used to characterize their conformation. Results from non-equilibrium simulations are compared to that of the equilibrium simulations (labelled as "pulled" and "norest"). The closed structure presented two states in equilibrium simulations, but just one state in the non-equilibrium simulations.

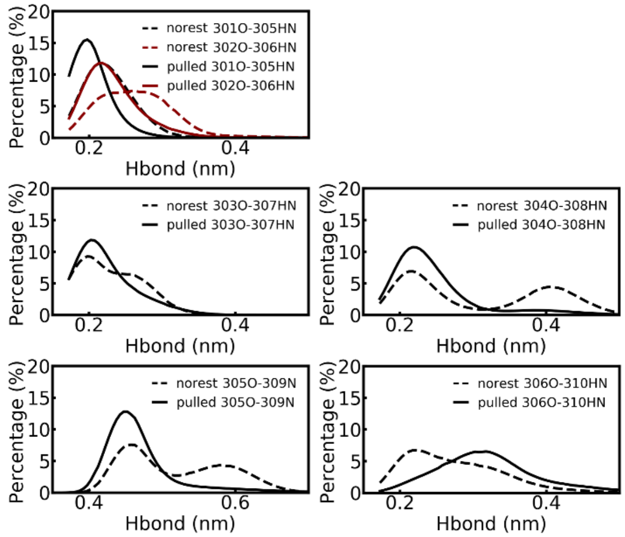

Supplementary Figure 14. Equilibrium simulations of the G302A and P309A mutants in the closed state. (a) Comparison of the conformations of the mutants and the wild type channel. The averaged structures of the mutants (cyan) are superimposed to that of the wild type (white). Cα atoms of res. 302, 309 are shown in space filling model and labelled as G302 and P309. (b) Ion currents and the numbers of waters in the cavity of the mutants. The results are shown as boxplot. The centre line, box limits, and whiskers represent the median, upper and lower quartiles, and 1.5 × interquartile range. The data points are shown in gray, while the averages and errors (standard error of the mean) are shown in red. n=10, n=10 and n=5 independent simulation replicas were conducted for the wild type, G302A and P309A mutants. (c) Free energy profiles for potassium ions and waters entering into the cavity. Positions of F304 and I308, as well as the position of Sc potassium binding site, are labelled by gray dashed lines. (d) Distributions of F304 side chain orientation. Results of the mutants are compared to those of the wild type in panels b-d.

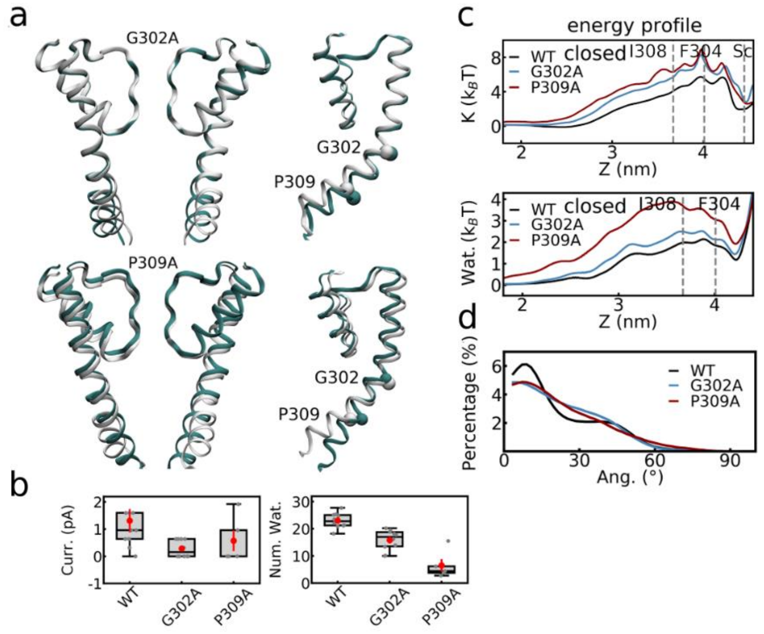

Supplementary Figure 15. Conformations of the inner TM helices of the G302A and P309A mutants in equilibrium simulations. Length distributions of the backbone hydrogen bonds were used to characterize the conformation. Results of the mutants are compared to those of the wild type.

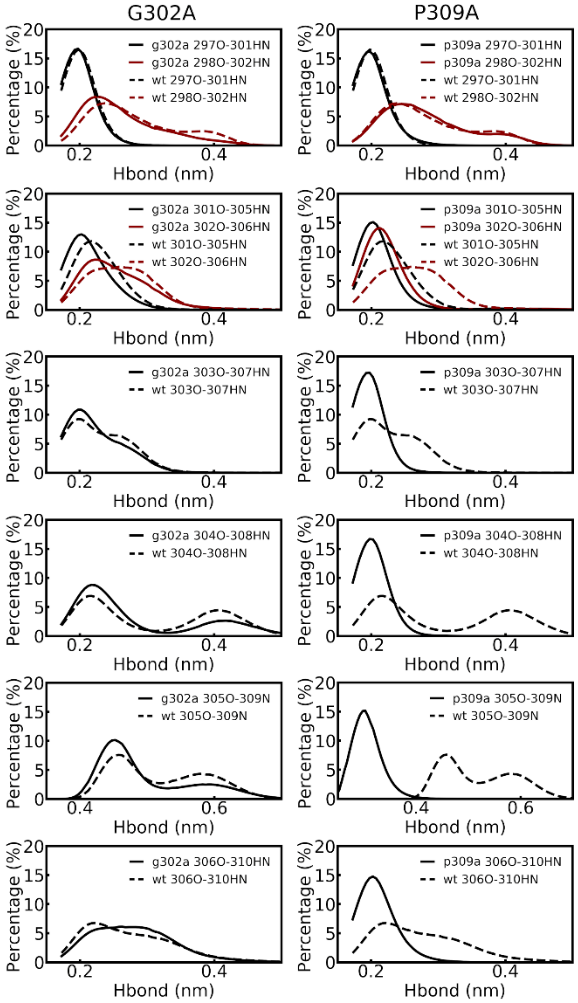

Supplementary Figure 16. Comparison of the closed structures obtained by enforced dehydration and equilibrium simulations for the G302A and P309A mutants. Average structures of the mutants for all replicas in non-equilibrium simulations (cyan) are aligned to the average structures for the equilibrium simulations (white). Top: G302A; Down: P309A. Res. 302 and 309 are labelled as G302 and P309, although they are actually “A302, P309” and “G302, A309” in these two mutants.

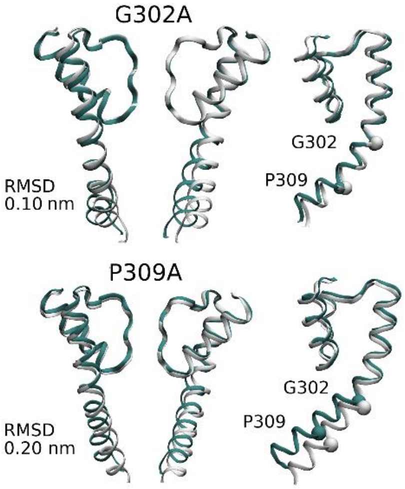

Supplementary Figure 17. Conformations of the inner TM helices of the G302A and P309A mutants obtained by enforced dehydration. Length distributions of the backbone hydrogen bonds were used to characterize the conformation. Results of the non-equilibrium simulations (labelled as "pulled") are compared to those of the equilibrium simulations (labelled as "norest").

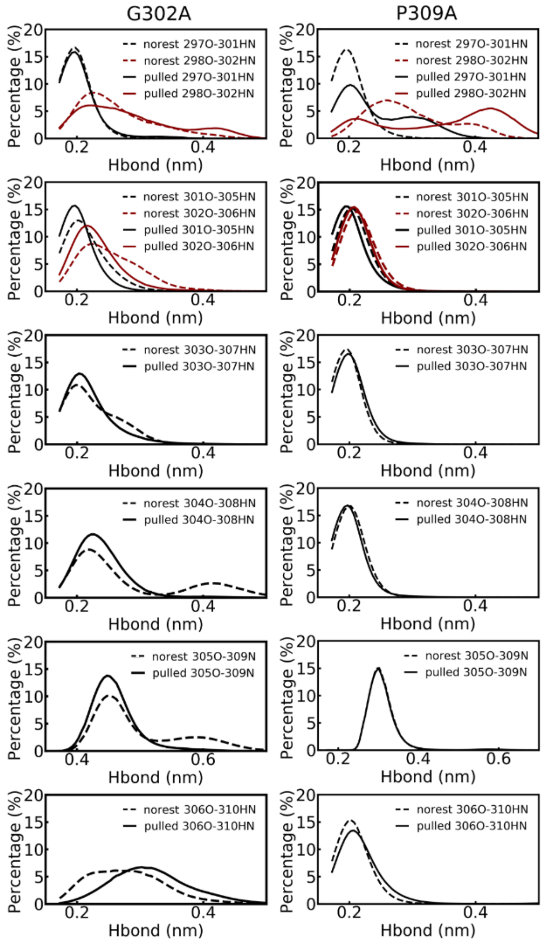

Supplementary Figure 18. The procedure of cavity dehydration in non-equilibrium simulations using dummy atoms (protocol 1). Snapshots of a representative trajectory are presented to show different hydration levels at different simulation times. The water oxygens and the F304 side chains are shown in red space filling model and in cyan stick model.

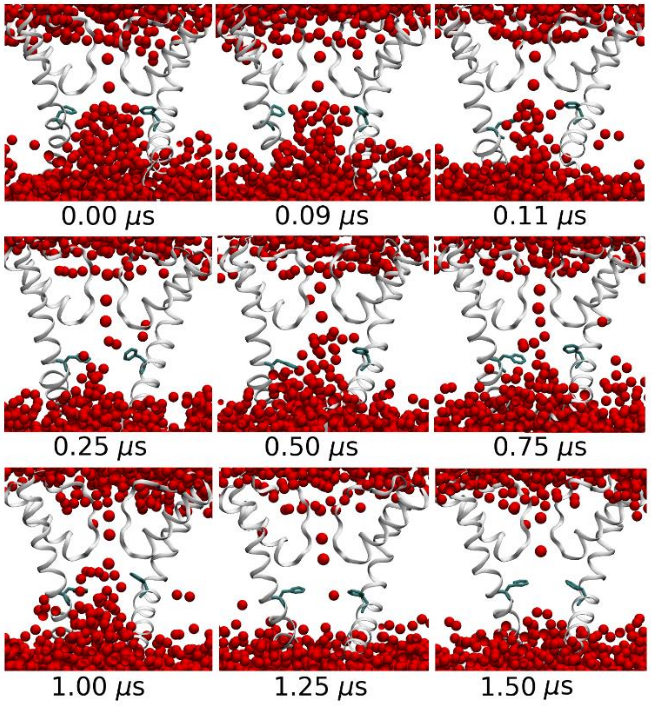

Supplementary Figure 19. Unwinding of the inner TM helices around V294 in non-equilibrium simulations using dummy atoms (protocol 1). Left: Length distributions of backbone hydrogen bonds at V294. Comparison between the equilibrium (no rest.) and non-equilibrium (prot. 1) simulations suggested an unwinding around V294. Right: Structural alignment of the unwound (cyan) and wounded (white) inner helix.

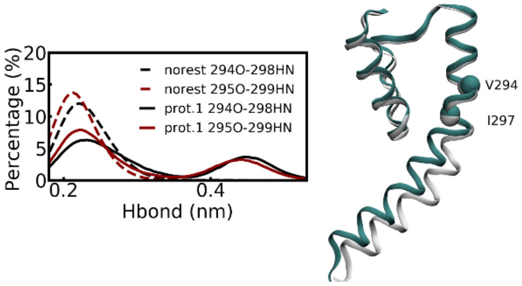

Supplementary Figure 20. Conformational transition of BK in a representative non-equilibrium simulation using dummy atoms (protocol 2). Water numbers in the cavity, RMSD values relative to the open and closed states, backbone hydrogen bonds at the kink (297O-301HN, and 298O-302HN), F304 Cα-Cα distance, and the F304 side chain orientation are shown as a function of simulation time. The lines were smoothed for clarity by averaging every 5 or 10 data points.

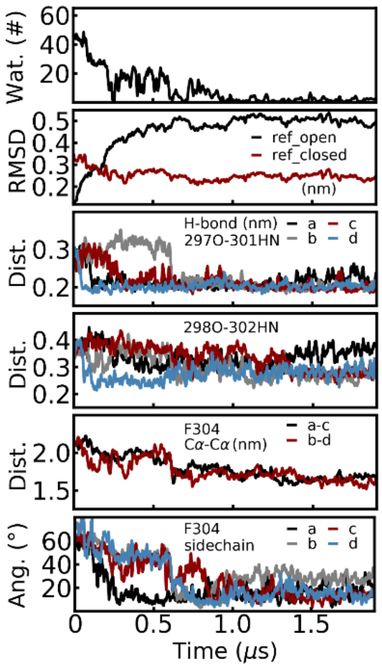

Supplementary Figure 21. Conformational transition from the closed to the open state induced by hydration of the central cavity. Results were presented for equilibrium simulations of the A305E mutant and non-equilibrium simulations of the wild type channel. (a) Comparison of the open conformations. The average structures of all of the A305E mutant simulations (top: 0.1-0.2 μs of equilibrium simulation, middle: 0.2-0.7 μs of the production simulation, cyan) and that of the enforced hydration simulations (cyan) are aligned to the average structure of the wild type (white). (b) Boxplot of the RMSD values and numbers of water molecules for each replica of the A305E mutant simulations (equi: 0.1-0.2 μs, prod: 0.2-0.7μs) and the wild type enforced hydration simulations. The centre line, box limits, and whiskers represent the median, upper and lower quartiles, and 1.5 × interquartile range. The data points are shown in gray dots, while the averages and errors (standard error of the mean, s.e.m.) are shown in red. n=5, n=10 and n=12 independent simulations were conducted for the equilibrium and production simulations of A305E mutant, and the wild type enforced hydration simulations. (c) Representative simulations of the A305E mutant (left) and the wild type channel (right). Number of water molecules in the cavity, RMSD values relative to the open and closed states, hydrogen bond length (297O-301HN, and 298O-302HN), F304 Cα-Cα distance, and F304 side chain orientation are shown as a function of simulation time. Note that the voltage was not applied for the first 0.2 μs (equi.) in the A305E simulations. In the enforced hydration simulations of the wild type channel, waters were restrained in the cavity for the first 0.5 μs (rest.), followed by another 0.5 μs simulations without any restraints ("equi."). The lines were smoothed for clarity by averaging every 5 or 10 data points. (d) Length distributions of the hydrogen bonds at the kink of the channel. We compared the results of the representative simulations in panel c with the equilibrium simulations of the wild type channel.

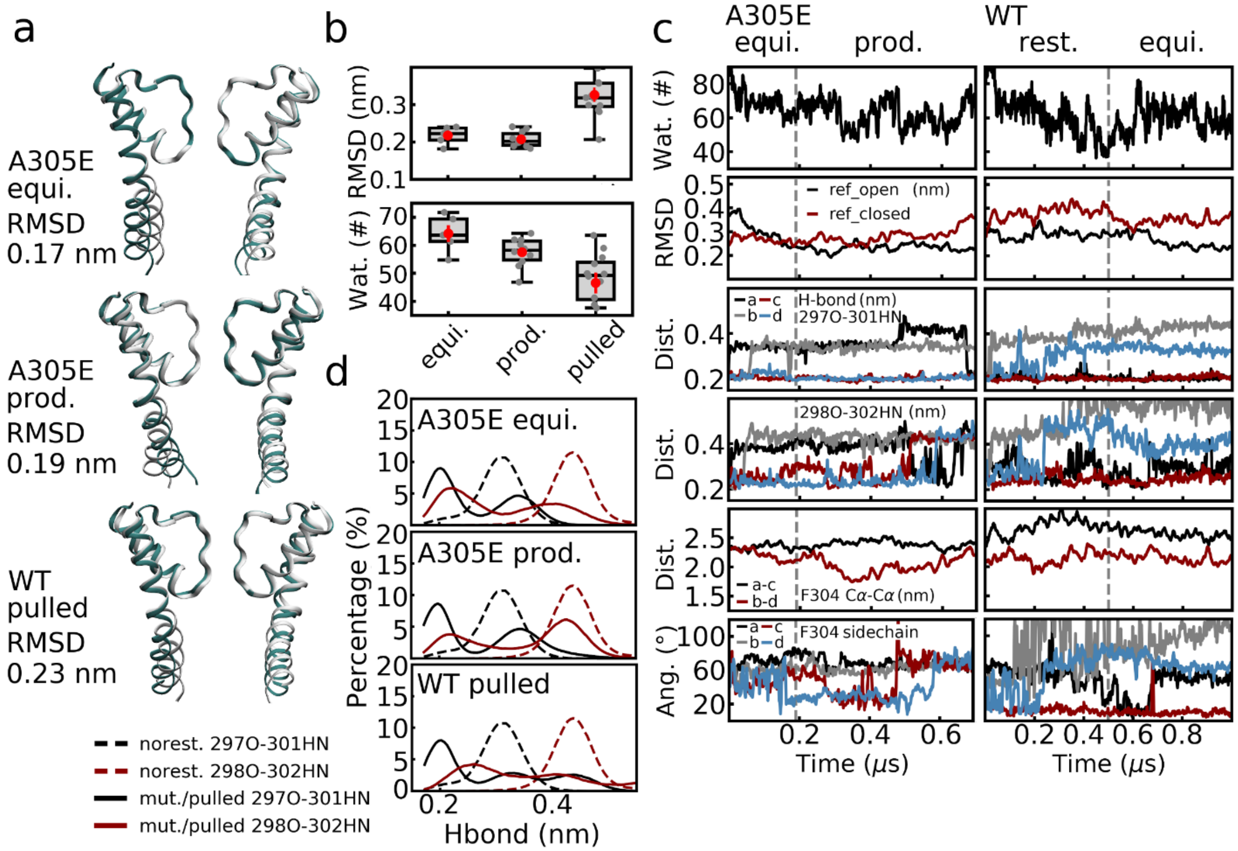
a
b
c
d
A305E equi. RMSD 0.17 nm
A305E prod. RMSD 0.19 nm
WT pulled RMSD 0.23 nm
RMSD (nm)
Wat. (#)
equi.
prod.
pulled
A305E equi.
A305E prod.
WT pulled
Percentage (%)
Hbond (nm)
norest. 297O-301HN
norest. 298O-302HN
mut./pulled 297O-301HN
mut./pulled 298O-302HN
A305E
equi. prod.
WT
rest. equi.
ref_open (nm)
ref_closed
H-bond (nm) 297O-301HN
298O-302HN (nm)
F304 Cα-Cα (nm)
F304 sidechain
Dist.
Ang. (°)
Time (μs)

Supplementary Figure 22. Conformational transition from the closed to the open state induced by hydration of the central cavity in simulations with backbone hydrogen bonds restrained. Backbone hydrogen bonds of the helices following the kink were restrained in these simulations (see Supplementary Table 2). We present the results of equilibrium simulations of the A305E mutant and the results of the non-equilibrium simulations of the wild type (referred as "pulled" in the figure). (a) Comparison of the open conformations. The average structure of all replicas of the A305E mutant simulations (left: 0.1-0.2 μs, cyan) and of the enforced hydration simulations (right: 0.75-1.0 μs, cyan) are aligned to the average structure of the wild type (white). Boxplot of the (b) RMSD values and (c) numbers of water molecules for the A305E mutant and wild type simulations. The centre line, box limits, and whiskers represent the median, upper and lower quartiles, and 1.5 × interquartile range. The data points are shown in gray dots, while the averages and errors (standard error of the mean, s.e.m.) are shown in red. Results of the A305E mutant and WT channel include 10 and 5 simulation replicas. (d) Representative simulations of the A305E mutant (left) and the wild type channel (right). The number of water molecules in the cavity, RMSD values relative to the open and closed states, hydrogen bond length (297O-301HN, and 298O-302HN), F304 Cα-Cα distance, and F304 side chain orientation are shown as a function of simulation time. The lines were smoothed for clarity by averaging every 5 or 10 data points. (e) Length distributions of the hydrogen bonds at the kink of the channel. We compared the results of the representative simulations in panel d with the equilibrium simulations of the wild type channel.

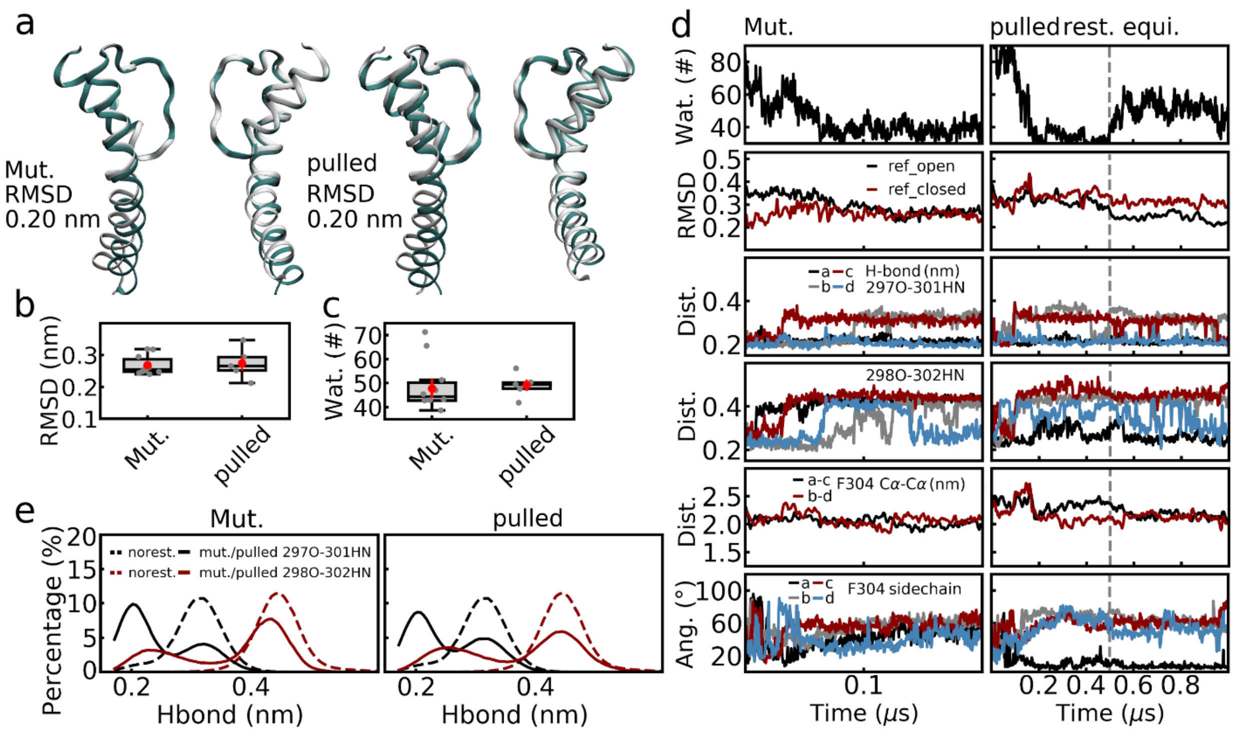
a
Mut.
RMSD
0.20 nm
pulled
RMSD
0.20 nm
b
RMSD (nm)
Mut.
pulled
c
Wat. (#)
Mut.
pulled
d
Mut.
pulled
rest. equi.
Wat. (#)
RMSD
ref_open
ref_closed
Dist.
a c H-bond (nm)
b d 297O-301HN
298O-302HN
a-c F304 Cα-Cα (nm)
b-d
Ang. (°)
a c F304 sidechain
b d
Time (μs)
e
Mut.
pulled
Percentage (%)
norest.
mut./pulled 297O-301HN
norest.
mut./pulled 298O-302HN
Hbond (nm)

Supplementary Figure 23. Intermediate state of MthK induced by non-equilibrium simulations. (a) Comparison of the open state (PDB entry: 3ldc [http://doi.org/10.2210/pdb3LDC/pdb]), the intermediate state (average structure obtained by enforced dehydration simulations), and the closed state (PDB entry: 6u6d [http://doi.org/10.2210/pdb6U6D/pdb]). Only two subunits are shown for clarity. F87 side chains are also shown in stick model. (b) Alignment of the three structures in panel a. (c) Definition of the helical bending angle. Cα atoms of V81 and G85 are also shown by space filling model. (d) Average water numbers in the cavity of the open state and the intermediate states obtained by two different non-equilibrium simulation methods. The centre line, box limits, and whiskers represent the median, upper and lower quartiles, and 1.5 × interquartile range. The data points are shown in gray dots, while the averages and errors (standard error of the mean, s.e.m.) are shown in red. n=10, n=4 and n=8 independent simulation replicas were conducted for the open state, the intermediate states obtained using chain reaction coordinates (pulled) and dummy atoms (dummy). (e) Comparison of the intermediate states obtained by two non-equilibrium methods. Distributions of backbone hydrogen bonds at the kink, helical bending angle, and the F87 side chain orientation are shown. Corresponding values of the open and closed states are labeled by solid and dashed lines in gray. (f) A representative simulation using the enforced cavity dehydration method. Water numbers in the cavity, RMSD values relative to the open and closed states, hydrogen bond length (81O-85HN), helical bending angle and Phe87 side chain orientation are shown as a function of simulation time. The lines were smoothed for clarity by averaging every 5 or 10 data points.

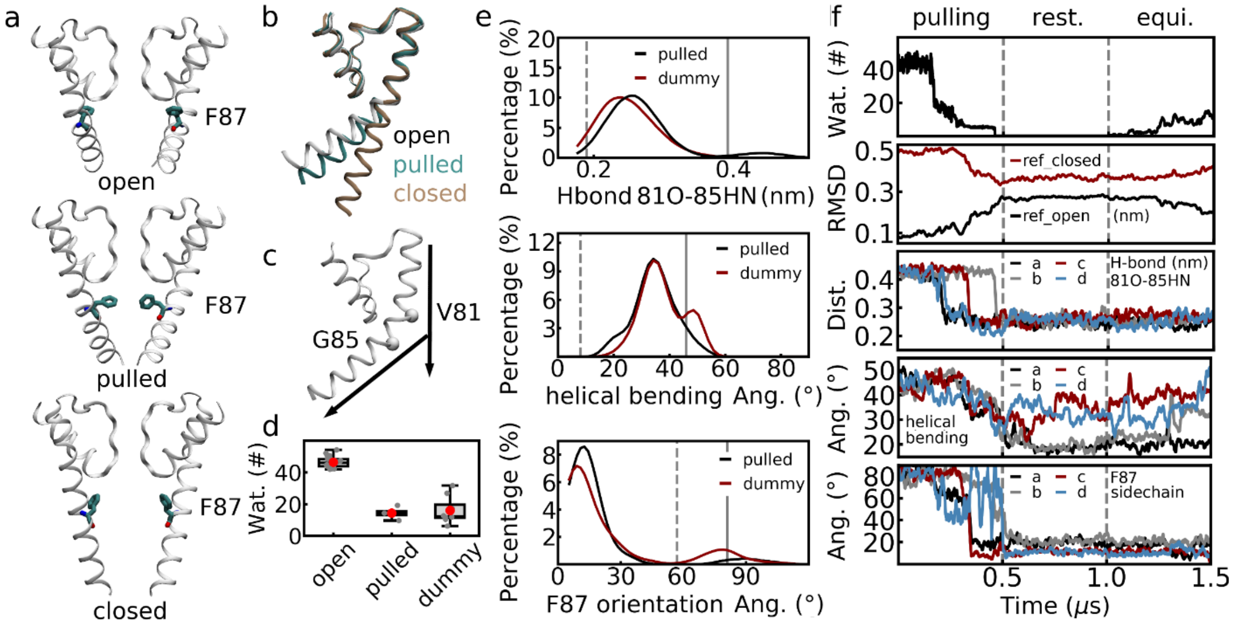

a
F87
open
F87
pulled
F87
closed
b
open
pulled
closed
c
V81
G85
d
Wat. (#)
open
pulled
dummy
e
Percentage (%)
pulled
dummy
Hbond 81O-85HN (nm)
helical bending Ang. (°)
F87 orientation Ang. (°)
f
pulling
rest.
equi.
Wat. (#)
RMSD
ref_closed
ref_open
(nm)
Dist.
H-bond (nm)
81O-85HN
Ang. (°)
helical bending
F87 sidechain
Time (μs)

Supplementary Figure 24. Conformational transition of MthK in a representative non-equilibrium simulation using dummy atoms. Water numbers in the cavity, RMSD values relative to the open and closed states, hydrogen bond length (81O-85HN), helical bending angle and F87 side chain orientation are shown as a function of simulation time. The lines were smoothed for clarity by averaging every 5 or 10 data points.

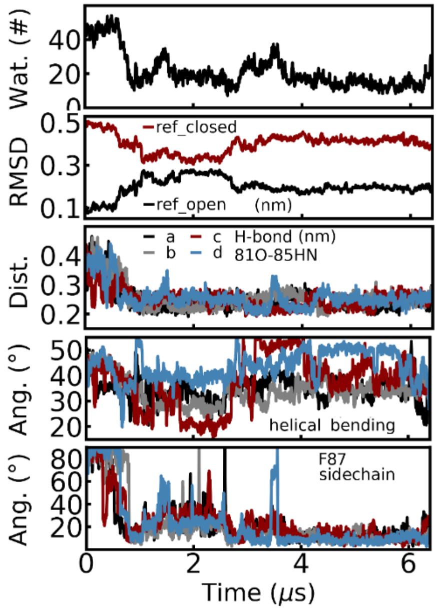

Supplementary Figure 25. Different orientations of glutamic acid residues in the TM helices of MthK. Structures of the open state, the closed state and the average structure obtained from non-equilibrium simulations (enforced dehydration) are compared. E92 and E96 are shown in stick model in cyan.

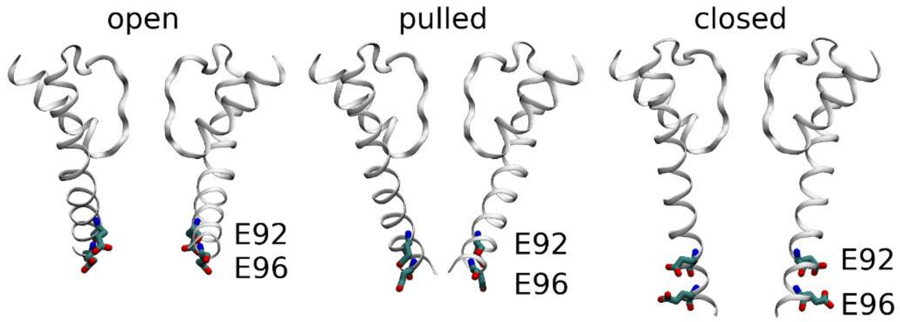

Supplementary Figure 26. **Lipid-protein interactions for the open and closed states of the aBK channel.** (a-d) Lipid distributions around and in the cavity of the open state channel. (a) Number of water molecules as a function of simulation time in a trajectory in which three lipids distributed in the cavity eventually dewetted the cavity completely. (b) Snapshots showing lipid distribution in the cavity at different time points labeled in panel a. The three lipids are shown in cyan, green and orange. (c) A lipid partially entered into the cavity in a trajectory. (d) Four lipids binding at the subunit interfaces with their polar headgroups pointing to the central cavity. (e-h) Different categories of lipid-protein interactions for the closed state of the channel. The inner TM helices of the protein are shown in gray, whereas the involved lipids are shown in space filling model.

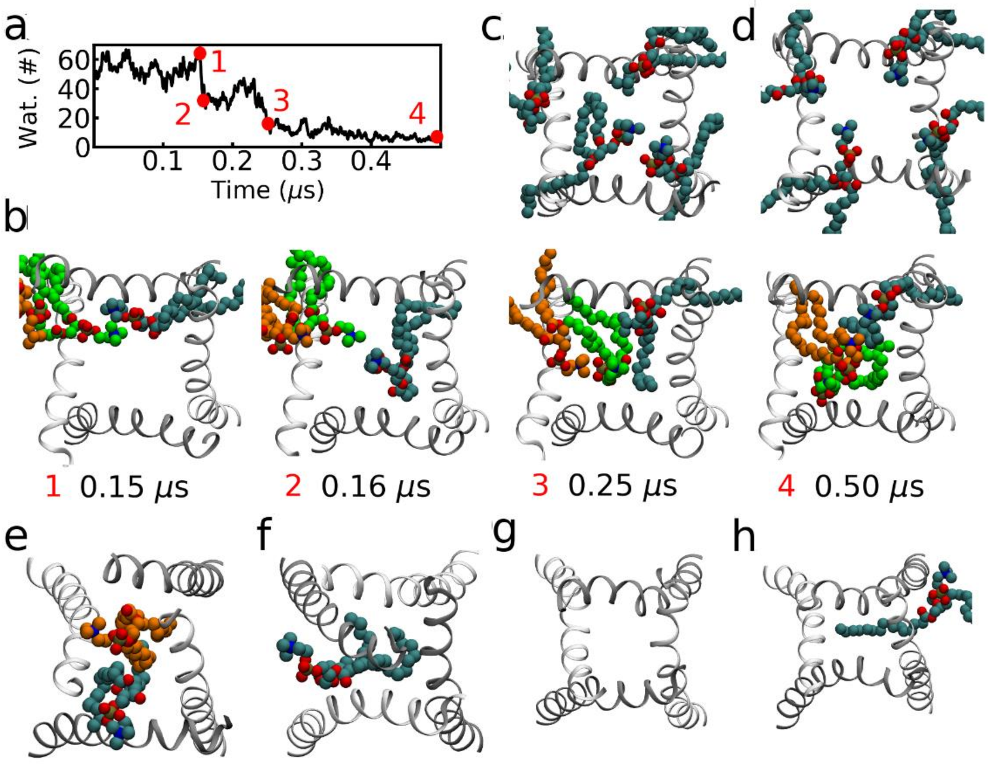

Supplementary Figure 27. Free energy profiles of potassium ions and water molecules entering into the cavity of the wild type channel. The results for simulations with and without using dummy atoms are compared.

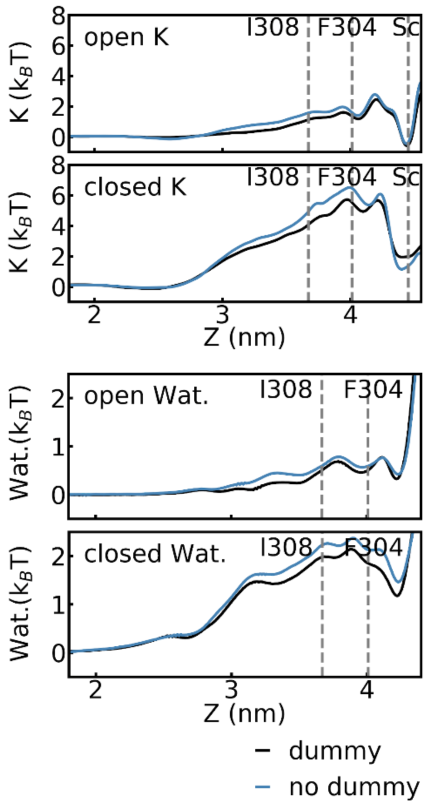

Supplementary References.

1. Fan, C. *et al.* Ball-and-chain inactivation in a calcium-gated potassium channel. *Nature* **580**, 288–293 (2020).
2. Gu, R. X. & de Groot, B. L. Lipid-protein interactions modulate the conformational equilibrium of a potassium channel. *Nat Commun* **11**, 2162 (2020).
3. Nose, S. A molecular dynamics method for simulations in the canonical ensemble. *Mol Phys* **52**, 255–268 (1983).
4. Hoover, W. G. Canonical dynamics: Equilibrium phase-space distributions. *Phys Rev A (Coll Park)* **31**, 1695–1697 (1985).
5. Parrinello, M. & Rahman, A. Polymorphic transitions in single crystals: A new molecular dynamics method. *J Appl Phys* **52**, 7182–7190 (1981).
6. Nose, S. & Klein, M. L. Constant pressure molecular dynamics for molecular systems. *Mol Phys* **50**, 1055–1076 (1983).
7. Steinbach, P. J. & Brooks, B. R. New spherical-cutoff methods for long-range forces in macromolecular simulation. *J Comput Chem* **15**, 667–683 (1994).
8. Darden, T., York, D. & Pedersen, L. Particle mesh Ewald: An N log(N) method for Ewald sums in large systems. *J Chem Phys* **98**, 10089–10092 (1993).
9. Essmann, U. *et al.* A smooth particle mesh Ewald method. *J Chem Phys* **103**, 8577–8592 (1995).
10. Hub, J. S. Joint reaction coordinate for computing the free-energy landscape of pore nucleation and pore expansion in lipid membranes. *J Chem Theory Comput* **17**, 1229–1239 (2021).
11. Hub, J. S. & Awasthi, N. Probing a continuous polar defect: A reaction coordinate for pore formation in lipid membranes. *J Chem Theory Comput* **13**, 2352–2366 (2017).
12. Kopec, W., Rothberg, B. S. & de Groot, B. L. Molecular mechanism of a potassium channel gating through activation gate-selectivity filter coupling. *Nat Commun* **10**, 5336 (2019).
