# Supplementary material for: Central cavity dehydration as a gating mechanism of potassium channels
Source: Nat Commun. 2023 Apr 17;14:2178. doi: 10.1038/s41467-023-37531-8 (PMC10110622; doi:10.1038/s41467-023-37531-8)
Supplement: Supplementary file 3 — Reporting Summary [file 41467_2023_37531_MOESM3_ESM.pdf]

Corresponding author(s): Bert L. de Groot

Last updated by author(s): Mar 1, 2023

Reporting Summary

Nature Portfolio wishes to improve the reproducibility of the work that we publish. This form provides structure for consistency and transparency in reporting. For further information on Nature Portfolio policies, see our Editorial Policies and the Editorial Policy Checklist.

Statistics

For all statistical analyses, confirm that the following items are present in the figure legend, table legend, main text, or Methods section.

n/a	Confirmed	
☐	☒	The exact sample size (*n*) for each experimental group/condition, given as a discrete number and unit of measurement
☒	☐	A statement on whether measurements were taken from distinct samples or whether the same sample was measured repeatedly
☒	☐	The statistical test(s) used AND whether they are one- or two-sided<br>*Only common tests should be described solely by name; describe more complex techniques in the Methods section.*
☒	☐	A description of all covariates tested
☒	☐	A description of any assumptions or corrections, such as tests of normality and adjustment for multiple comparisons
☐	☒	A full description of the statistical parameters including central tendency (e.g. means) or other basic estimates (e.g. regression coefficient) AND variation (e.g. standard deviation) or associated estimates of uncertainty (e.g. confidence intervals)
☒	☐	For null hypothesis testing, the test statistic (e.g. *F*, *t*, *r*) with confidence intervals, effect sizes, degrees of freedom and *P* value noted<br>*Give P values as exact values whenever suitable.*
☒	☐	For Bayesian analysis, information on the choice of priors and Markov chain Monte Carlo settings
☒	☐	For hierarchical and complex designs, identification of the appropriate level for tests and full reporting of outcomes
☒	☐	Estimates of effect sizes (e.g. Cohen's *d*, Pearson's *r*), indicating how they were calculated

Our web collection on statistics for biologists contains articles on many of the points above.

Software and code

Policy information about availability of computer code

Data collection	GROMACS2019/2020, CHARMM36m force field, Fortran code (Kopec et al., Nature Communications, 10, 5366, 2019)
Data analysis	Python scripts (python version 2.7) to calculate orientations of Phe304 sidechains, numbers of water molecules and potassium ions in the cavity, numbers of water molecules in the solvation shells of potassium ions, the free energy profiles of water molecules and potassium ions are available from https://doi.org/10.6084/m9.figshare.21803673.v1

For manuscripts utilizing custom algorithms or software that are central to the research but not yet described in published literature, software must be made available to editors and reviewers. We strongly encourage code deposition in a community repository (e.g. GitHub). See the Nature Portfolio guidelines for submitting code & software for further information.

Data

Policy information about availability of data

All manuscripts must include a data availability statement. This statement should provide the following information, where applicable:

- Accession codes, unique identifiers, or web links for publicly available datasets
- A description of any restrictions on data availability
- For clinical datasets or third party data, please ensure that the statement adheres to our policy

The experimental protein structures used in this work are available form the protein data bank (PDB entry: 5tj6 [http://doi.org/10.2210/pdb5TJ6/pdb], 5tji [http://doi.org/10.2210/pdb5TJI/pdb], 3ldc [http://doi.org/10.2210/pdb3LDC/pdb], 6u6d [http://doi.org/10.2210/pdb6U6D/pdb]).The data generated from MD simulations

in this study underlying Figs 2d-g, 3b, 4b-e, 5b-d, Supplementary Tables 1, 3-4, and Supplementary Figs. 4, 5b, 6, 8-9, 10a-d, 11, 12b-d, 13, 14b-d, 15, 17, 20, 21b-d, 22b-e, 23d-f, 24, 27, as well as the initial and final conformations of the MD simulations have been deposited to the Figshare database and are available from https://doi.org/10.6084/m9.figshare.22194646.v1

Human research participants

Policy information about studies involving human research participants and Sex and Gender in Research.

Reporting on sex and gender	not appliable. This work only involves computer simulations of biological molecules, does not involve human participants.
Population characteristics	not appliable. This work only involves computer simulations of biological molecules, does not involve human participants.
Recruitment	not appliable. This work only involves computer simulations of biological molecules, does not involve human participants.
Ethics oversight	not appliable. This work only involves computer simulations of biological molecules, does not involve human participants.

Note that full information on the approval of the study protocol must also be provided in the manuscript.

Field-specific reporting

Please select the one below that is the best fit for your research. If you are not sure, read the appropriate sections before making your selection.

☒ Life sciences ☐ Behavioural & social sciences ☐ Ecological, evolutionary & environmental sciences

For a reference copy of the document with all sections, see nature.com/documents/nr-reporting-summary-flat.pdf

Life sciences study design

All studies must disclose on these points even when the disclosure is negative.

Sample size	We increase the sample size (the number of simulation replicas) as much as we can, to ensure statistical reliability of our results.
Data exclusions	We do not exclude any data.
Replication	For each simulation system, we conducted 5-20 simulation replicas and calculated the mean and standard error of the mean to measure the statistical significance and to make sure the results are reproducible . We presented the averages and representative cases of our simulations, in our manuscript.
Randomization	In the MD simulations of this work, we assign different initial velocities for the parallel simulations in the same group to ensure sufficient sampling. We do not need to allocate samples/organisms/participants to groups. In this regard, this is not relevant in our situation.
Blinding	As mentioned above, in the MD simulations in this work, we do not need to allocate samples/organisms/participants to groups. We only need to setup the initial state and the simulation parameters. Besides, once the simulations are done, the data are transparent to the analyzer, i.e., we know which trajectory is from which simulation. In this regard, blinding is not relevant in our case.

Reporting for specific materials, systems and methods

We require information from authors about some types of materials, experimental systems and methods used in many studies. Here, indicate whether each material, system or method listed is relevant to your study. If you are not sure if a list item applies to your research, read the appropriate section before selecting a response.

Materials & experimental systems

n/a	Involved in the study	
☒	☐	Antibodies
☒	☐	Eukaryotic cell lines
☒	☐	Palaeontology and archaeology
☒	☐	Animals and other organisms
☒	☐	Clinical data
☒	☐	Dual use research of concern

Methods

n/a	Involved in the study	
☒	☐	ChIP-seq
☒	☐	Flow cytometry
☒	☐	MRI-based neuroimaging
